# New activation mechanism for half-sandwich organometallic anticancer complexes†
†Electronic supplementary information (ESI) available. CCDC 1420046–1420052 and 1569283. For ESI and crystallographic data in CIF or other electronic format see DOI: 10.1039/c7sc05058e


**DOI:** 10.1039/c7sc05058e

**Published:** 2018-03-01

**Authors:** Samya Banerjee, Joan J. Soldevila-Barreda, Juliusz A. Wolny, Christopher A. Wootton, Abraha Habtemariam, Isolda Romero-Canelón, Feng Chen, Guy J. Clarkson, Ivan Prokes, Lijiang Song, Peter B. O'Connor, Volker Schünemann, Peter J. Sadler

**Affiliations:** a Department of Chemistry , University of Warwick , Gibbet Hill Road , Coventry CV4 7AL , UK . Email: P.J.Sadler@warwick.ac.uk; b Department of Physics , University of Kaiserslautern , Erwin-Schrödinger-Straße 46 , 67663 Kaiserslautern , Germany

## Abstract

The Cp^*x*^ C–H protons in certain organometallic Rh^III^ half-sandwich anticancer complexes [(η^5^-Cp^*x*^)Rh(*N*,*N*′)Cl]^+^, where Cp^*x*^ = Cp*, phenyl or biphenyl-Me_4_Cp, and *N*,*N*′ = bipyridine, dimethylbipyridine, or phenanthroline, can undergo rapid sequential deuteration of all 15 Cp* methyl protons in aqueous media at ambient temperature. DFT calculations suggest a mechanism involving abstraction of a Cp* proton by the Rh–hydroxido complex, followed by sequential H/D exchange, with the Cp* rings behaving like dynamic molecular ‘twisters’. The calculations reveal the crucial role of p_π_ orbitals of *N*,*N*′-chelated ligands in stabilizing deprotonated Cp^*x*^ ligands, and also the accessibility of Rh^I^–fulvene intermediates. They also provide insight into why biologically-inactive complexes such as [(Cp*)Rh^III^(en)Cl]^+^ and [(Cp*)Ir^III^(bpy)Cl]^+^ do not have activated Cp* rings. The thiol tripeptide glutathione (γ-l-Glu-l-Cys-Gly, GSH) and the activated dienophile *N*-methylmaleimide, (NMM) did not undergo addition reactions with the proposed Rh^I^–fulvene, although they were able to control the extent of Cp* deuteration. We readily trapped and characterized Rh^I^–fulvene intermediates by Diels–Alder [4+2] cyclo-addition reactions with the natural biological dienes isoprene and conjugated (9*Z*,11*E*)-linoleic acid in aqueous media, including cell culture medium, the first report of a Diels–Alder reaction of a metal-bound fulvene in aqueous solution. These findings will introduce new concepts into the design of organometallic Cp* anticancer complexes with novel mechanisms of action.

## Abstract 2

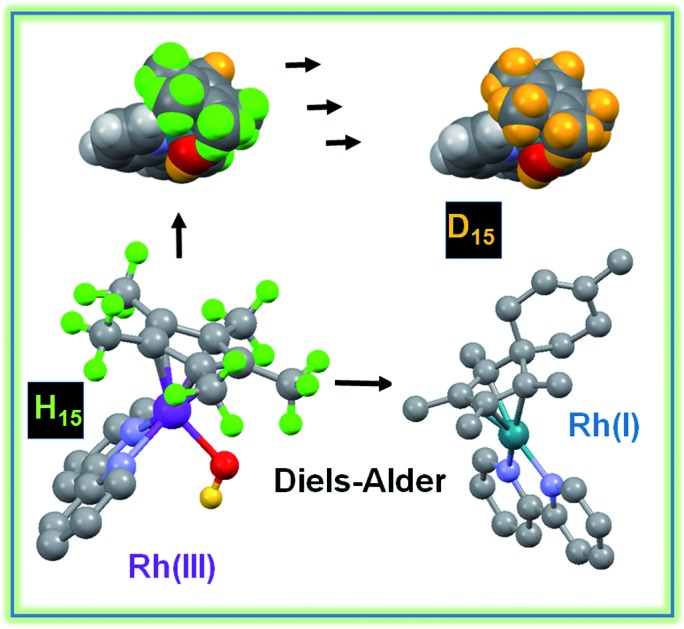
Half-sandwich Rh^III^ anticancer complexes with activated Cp* rings not only undergo sequential CH_3_ H–D exchange, but also react with biological dienes, generating Rh^I^ Diels–Alder adducts in aqueous media at ambient temperature.

## Introduction

Methylated cyclopentadienyl ligands, and especially pentamethyl-cyclopentadienyl (Cp*), are often used as η^5^–π-bonded ligands in transition metal complexes.^1^,^2^ Cp* and substituted Cp* (Cp^*x*^, *e.g.* tetramethyl(biphenyl)cyclopentadienyl, Cp^*x*PhPh^) ligands form highly stable complexes with, for example, the low-spin d^6^ metal ions Rh^III^ and Ir^III^.^3^–^6^ Such Rh^III^ and Ir^III^ complexes are of interest for their wide range of properties, including activity as catalysts and anticancer agents,^3^,^4^,^7^–^12^ and sometimes as both.^13^,^14^

The biologically-active centre in half-sandwich [(η^5^-Cp^*x*^)Rh/Ir(*N*,*N*′/*C*,*N*)Cl] anticancer complexes containing *N*,*N*′- or *C*,*N*-chelated ligands is usually thought to be the metal ion itself.^10^–^14^ Initial displacement of chloride by water (aquation) can facilitate binding to target biomolecules, the possibility of direct reactions involving the Cp^*x*^ ligand under biologically-relevant conditions has yet to be investigated.

In general C–H bonds in hydrocarbons are very stable and difficult to activate. They are only weakly acidic, and the available common bases are not strong enough.^15^,^16^ Such C–H bond activation can be promoted especially by the electronic effects of platinum group metals, and numerous examples have now been reported.^17^–^21^ However, reported Cp^*x*^ activation reactions usually require harsh conditions.

In an early example, Kang and Maitlis deuterated the methyl groups in the dinuclear Rh^III^ complex [(Cp*)Rh(μ^2^-OH)_3_Rh(Cp*)]Cl in D_2_O after addition of OD^–^, but the process was slow, requiring over 3 days at the elevated temperature of 343 K.^22^ At a similar time, Bercaw *et al.* used very strong bases in non-polar solvents to synthesize Me_4_CpCH_2_–bridged Ti^II^ complexes. These were sensitive to traces of water.^23^ Wei *et al.* have shown that deuteration of the Cp* in [(Cp*)RhD_2_(BPin)] (BPin = bis(pinacolato)diboron) occurs readily in MeOD-*d*_4_ and synthesized the deuterated compound [(D_15_Cp*)RhD_2_(BPin)], under reflux in D_2_O/OD^–^ for 3 days.^24^ Ciancaleoni *et al.* showed that deuteration of [(Cp*)RhCl(PTA)_2_]^+^ (PTA = 7-phospha-1,3,5-triaza-adamantane) is assisted by the basic centers in the PTA ligand,^25^ and proposed a mechanism in which a Rh–OH species deprotonates the Cp* ring followed by formation of a Rh^I^–fulvene species, but no experimental evidence for the fulvene complex was obtained.

Quintana *et al.*^26^ and Pitman *et al.*^27^ previously reported the direct involvement of Cp* ligands in hydride transfer reactions. In the former case, initial protonation of Cp* in the Rh^I^ catalyst [(Cp*)Rh(bpy)] can lead to formation of H_2_ in dry acetonitrile. In the latter case, [(Cp*H)Rh(bpy)Cl], generated by reduction of the Rh^III^ complex [(Cp*)Rh(bpy)Cl]^+^ followed by acidification in ether, was isolated, and shown to reduce NAD^+^ to NADH. The Cp* methyl protons underwent exchange with deuterium. Peng *et al.* have recently characterized the Rh^I^ phenanthroline analogue containing protonated Cp*, [(Cp*H)Rh(phen)Cl].^28^ A recent report by Blakemore *et al.*^29^ introduced Cp*Rh complexes of substituted 2,2′-bipyridyl as effective hydrogen evolution catalysts involving the formation of unusual η^4^-pentamethylcyclopentadiene–Rh^I^ intermediates under protic conditions.

Our own investigations began with observations that in aqueous solutions at ambient temperature, methyl ^1^H NMR peaks of some [(Cp*)RhCl(*N*,*N*′)Cl]^+^ complexes which we had synthesized as potential anticancer agents and transfer hydrogenation catalysts, rapidly lost intensity, suggesting activation towards H/D exchange under mild conditions. We have investigated the conditions under which this occurs, including the influence of solvent, aquation, and pH, and monitored the reactions by both ^1^H NMR spectroscopy and high resolution FT ICR MS spectrometry. We have also investigated the influence of substituents on the Cp* ligand (Cp* *versus* Cp^*x*Ph^ and Cp^*x*PhPh^), influence of the *N*,*N*′-chelating ligand (aliphatic ethylenediamine (en) *versus* aromatic diamines bipyridyl (bpy), 4,4′-dimethyl-2,2′-bpyridine (mbpy), and phenanthroline (phen)), as well as the metal ion (Rh^III^*versus* Ir^III^). The X-ray crystal structures of 8 new complexes are reported.

**Chart 1:**
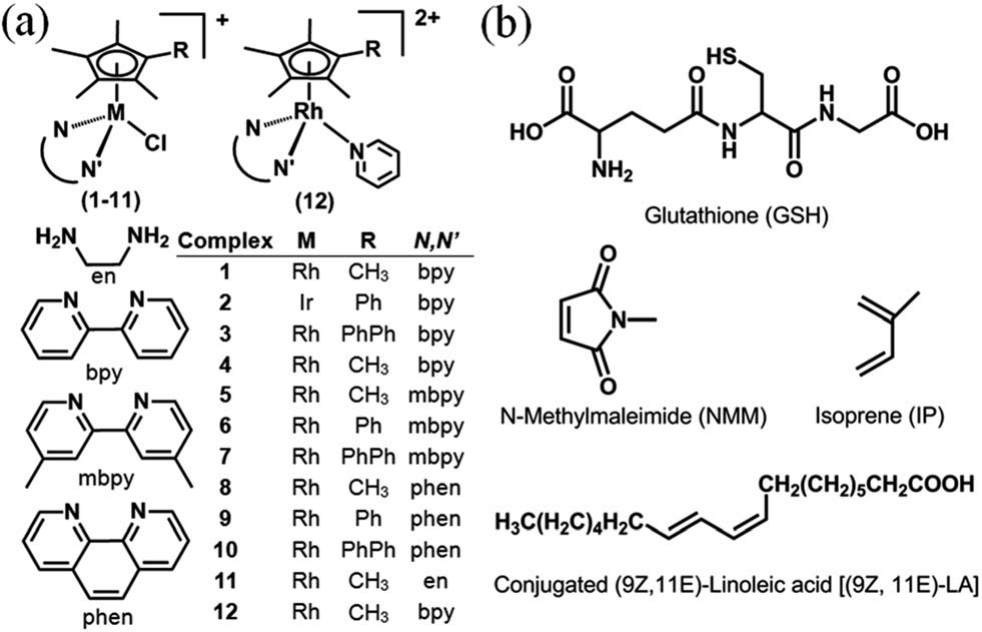
(a) Rh^III^ and Ir^III^ complexes studied here as PF_6_^–^ salts. (b) Structures of glutathione, dienes and dienophile used in attempts to trap fulvene intermediates.

We have used density functional theory (DFT) calculations to elucidate the mechanism of these Cp^*x*^ C–H activation reactions. Importantly the DFT calculations led to the prediction of accessible Rh^I^–fulvene states and so we investigated the trapping of such intermediates by addition reactions with thiols (glutathione), dienes (*N*-methyl maleimide, NMM) and dienophiles, Chart 1. The thiol tripeptide glutathione (γ-l-Glu-l-Cys-Gly) is present in cells at millimolar concentrations, and might be expected to undergo addition reactions with double bonds such as those in a fulvene, as might NMM which contains an activated double bond. Fulvene is known to undergo Diels–Alder reactions with both dienes and dienophiles.^30^ The exocyclic double bond of fulvene can also act as a dienophile in a Diels–Alder reaction.^30^,^31^ The presence of substituents on the exocyclic double bond shifts the Diels–Alder reaction to the endocyclic double bond.^30^,^31^ In attempts to trap adducts of the proposed fulvene intermediate as [4+2] cyclo-addition Diels–Alder adducts, we chose the natural biological conjugated dienes isoprene and (9*Z*,11*E*)-linoleic acid (Chart 1). Isoprene is the most abundant hydrocarbon in human breath, with an estimated human production of 17 mg per day.^32^,^33^ (9*Z*,11*E*)-Linoleic acid is a common dietary conjugated fatty acid, of particular interest for its potential role in the prevention and treatment of a variety of diseases,^34^ including roles in modulating the expression of oncogenes and cell cycle regulators.^34^,^35^

The surprising success of these Rh^I^–fulvene trapping reactions under mild conditions of biological relevance is likely to open up new avenues of investigation into reactions of this class of half-sandwich organometallic complexes and influence their design as biologically-active agents. Our discovery of Diels–Alder reactions of a metal-bound fulvene under mild conditions in aqueous solutions, appears to be unprecedented.

## Results and discussion

### Synthesis and characterization of complexes

Organo–Rh^III^ complexes **1** and **3–12** (Chart 1(a)) and Ir^III^ complex **2** containing Cp*, Cp^*x*Ph^ or Cp^*x*PhPh^, together with an *N*,*N*′-chelated ligand (en, bpy, mbpy or phen), and a monodentate chloride ligand were synthesized by the reaction of the appropriate chloride-bridged dimer and chelating ligand, except complex **12** which was prepared by substitution of chloride in complex **1** by pyridine. They were fully characterized by ^1^H NMR spectroscopy, ESI-mass spectrometry, and elemental analysis (details in the ESI†). The X-ray crystal structures of complexes **1**, **3**, **4**, **6**, **7**, **9**, **10** and **12** were determined (Fig. S1, Tables S1 and S2†). All exhibit the typical ‘piano-stool’ geometry. Rh–Cl distances are in the range of 2.385–2.403 Å, with complexes containing extended Cp^*x*^ rings having slightly longer bond lengths. The Rh–Cp^*x*^ centroid distance of 1.778–1.795 Å shows little change with extension of the Cp* ring. Interestingly, complexes **6**, **7** and **9** have one Rh–N distance slightly longer than the other, which might be due to the combined effects of crystal packing and ligand substituents. The bond lengths and angles for complexes **6** and **7** are similar to those reported for [(Cp*)Rh(phen)Cl]SO_3_CF_3_,^36^ and for complexes **1**, **3** and **4** are similar to those reported for [(Cp*)Rh(bpy)Cl]SO_3_CF_3_.^36^ For the pyridine complex **12**, the Rh–N(pyridine) bond (2.127(4) Å) is longer than other two Rh–N (bipyridine) bonds (*ca.* 2.105 Å).

### Hydrolysis

Aquation of chloride complexes **1** and **3–11** in 20% methanol-*d*_4_/80% D_2_O (1.4 mM, 310 K) was confirmed by comparing ^1^H NMR spectra before and after removal of the chloride ligand by reaction with AgNO_3_. Complexes **1**, **3–11** show fast hydrolysis at 310 K, reaching equilibrium in <10 min. Complete conversion of chloride complex **11** to the aqua adduct was observed, whereas **1**, and **3–10** reached equilibrium with 30–60% formation of the aqua species (Table S3†). 
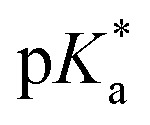
 values of the aqua adducts of **1**, **8**, **11** were determined by ^1^H NMR. The 
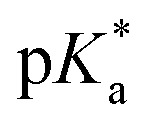
 values for the Rh^III^ complexes of 8.8–9.4 (Table S3†) are, *ca.* 1–2 p*K*_a_ units higher than for the Ir^III^ analogue **2**.^13^ The pyridine complex **12** showed only *ca.* 10% hydrolysis even after 10 days under similar experimental conditions.

**Fig. 1:**
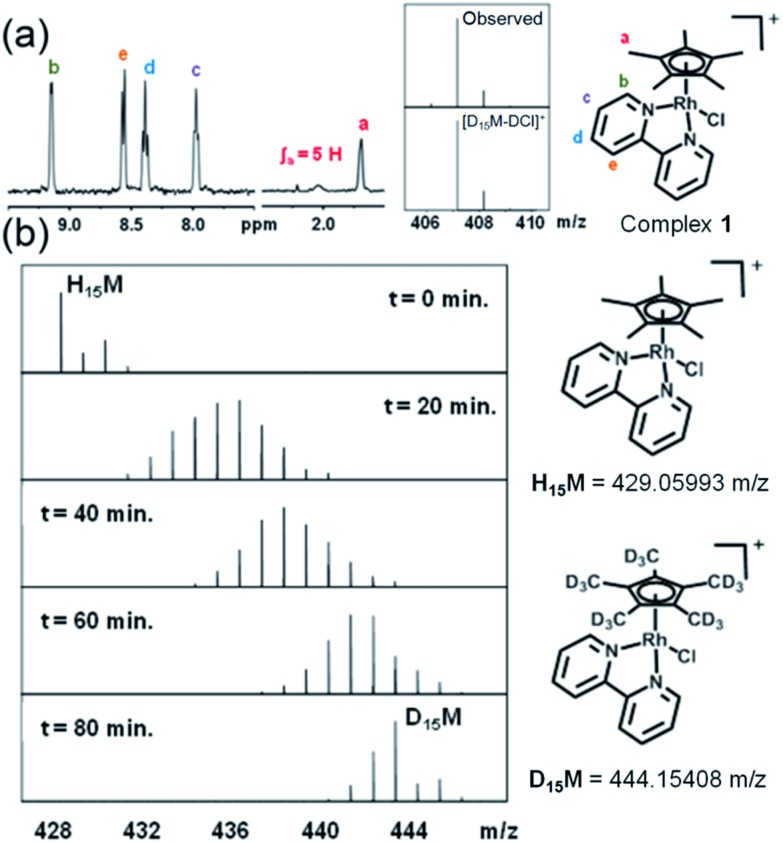
Sequential deuteration of complex **1** monitored by NMR and MS. (a) ^1^H NMR spectrum of **1** after reaction with AgNO_3_ in 60% MeOD-*d*_4_/40% D_2_O for 24 h, and FT-ICR mass spectrum after 72 h. (b) FT-ICR mass spectra of **1** treated with 0.95 mol equiv. AgNO_3_ and incubated at ambient temperature in 60% MeOD-*d*_4_/40% D_2_O. Isotopic pattern at *t* = 0 min corresponds to **1**, and at *t* = 20, 40, 60 and 80 min to the sum of isotopic patterns for **1** at various stages of deuteration.

When rhodium complexes **1** and **3–10** were dissolved in 60% MeOD-*d*_4_/40% D_2_O and reacted with AgNO_3_ to generate the corresponding aqua adducts, the ^1^H NMR spectra after 24 h at 310 K showed the expected peaks for the chelated ligands (bipyridine, phenanthroline or 4,4′-dimethyl-2,2′-bipyridine), but resonances corresponding to the methyl protons in the Cp*, Cp^*x*Ph^ and Cp^*x*PhPh^ ligands were not observed as the expected sharp singlets, but as broad signals, difficult to distinguish from the baseline (Fig. 1a, S2†). To identify the species formed, the FT-ICR MS spectrum of each product was recorded after 72 h (Fig. 1a). The MS peaks were assignable to the deuterated-Cp^*x*^ forms of complexes **1** and **3–10**. Hence, facile deuteration of the cyclopentadienyl ligands of these complexes occurs under very mild conditions. Notably however, we observed no deuteration of Ir–bpy complex **2**, nor Rh–en complex **11** (Fig. S3†).

### Sequential deuteration

Experiments were then performed to investigate the conditions under which deuteration occurs, in particular the effect of time, pH, solvent, hydrolysis and use of Ag^I^ to remove the Cl^–^ ligand. ^1^H NMR spectra of a 1.4 mM solution of complex **1** in 60% MeOD-*d*_4_/40% D_2_O treated with Ag^I^ were recorded at ambient temperature after 50 min and then every 410 s. A gradual decrease in intensity, with splitting and broadening of the Cp^*x*^ methyl peaks was observed. FT-ICR MS spectra were recorded every 10 min for 80 min and then after 27 and 42 h. Gradual deuteration of the Cp^*x*^ ring methyls was observed and it was evident that deuteration occurs *via* sequential replacement of methyl protons by deuterium from D_1_ to D_15_, complete deuteration (Fig. 1b).

Deuteration of Cp* ring methyls of **1** occurred both in the presence and absence of Ag^I^ (Fig. S4†). In the absence of Ag^I^, 30–50% hydrolysis was observed as a fast exchange on the NMR timescale. Addition of chloride suppressed both the hydrolysis and deuteration (Fig. S5†). Hence, we concluded that the formation of the Rh–aqua adduct is crucial for the deuteration.

The presence of methanol increased the rate of deuteration of **1** (Fig. S6†), increasing from 6% in D_2_O, to 20% in 40% D_2_O/60% MeOD-*d*_4_ to 66% in MeOD-*d*_4_ alone, after *ca.* 19 h at 293 K. Further evidence for involvement of the Rh–aqua complex in ring methyl deuteration was provided by [η^5^-(Cp*)Rh(bpy)(py)](PF_6_)_2_ (**12**). The X-ray crystal structure (Fig. S1, Table S2†) shows a strong Rh–N(pyridine) bond (2.127(4) Å), and even after 10 days at 310 K in 60% MeOD-*d*_4_/40% D_2_O, only 2 D had been incorporated into the Cp*, attributable to a small amount of aqua complex formed (Fig. S7†).

Since deprotonation of Rh–OH_2_ can provide a strategically-placed base for proton abstraction from Cp*, the effect of pH was investigated (Fig. S8†). Complex **1** in 60% MeOD-*d*_4_/40% D_2_O was treated with NaOD (in absence of Ag^I^). Quantitative full deuteration was achieved at ambient temperature by the time the first ^1^H NMR spectrum was recorded (*ca.* 12 min). In contrast, when the sample of **1** was made acidic with DNO_3_, no deuteration was observed after 12 h. These results are compatible with Rh-OD acting as a base for proton abstraction and subsequent deuteration of the Cp*.

### Back exchange

In order to study the reversibility of the deuterium/proton exchange, we prepared fully deuterated [(D_15_η^5^-Cp*)Rh(bpy)Cl]PF_6_ (1D_15_) and used ^2^H NMR to show that, as expected, back D/H exchange was slow (*ca.* 50%) after 3 days in 60% MeOH/40% H_2_O (Fig. S9†). Such facile deuterium labelling allows a wide range of reactions to be studied by ^2^H NMR, *e.g.* reaction with biologically-important molecules such as the tripeptide GSH or stability in complicated biological media such as cell culture medium, where ^1^H NMR peaks are heavily overlapped^37^ (exemplified in Fig. S9†).

### DFT calculations on H/D exchange

**Fig. 2:**
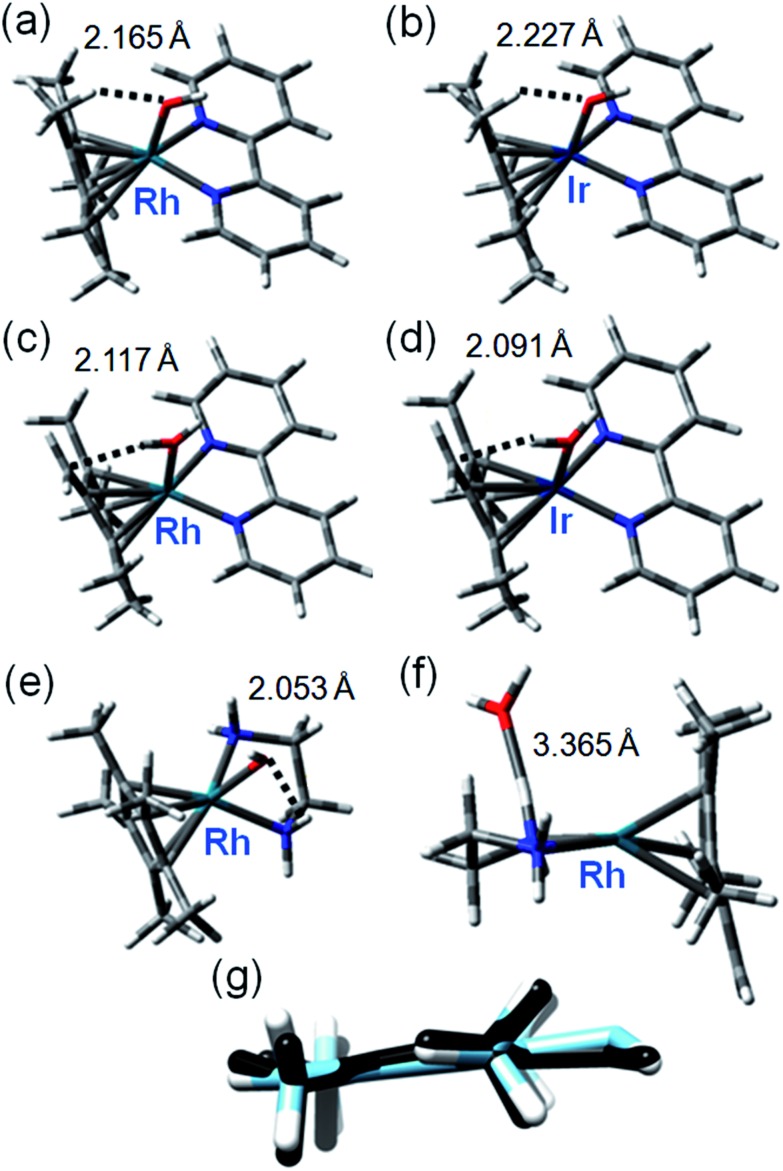
Key structures of hydroxo and aqua/fulvene complexes from DFT calculations. Optimized geometries with important weak interactions are shown. Calculated data for Ir^III^ and Rh^III^ systems are compared in Table S4,† and superposition of relevant OH and H_2_O complexes in Fig. S10.† (a) [Rh(Cp*)(bpy)OH]; (b) [Ir(Cp*)(bpy)OH]; (c) [Rh(Me_4_Cp=CH_2_)(bpy)OH_2_]; (d) [Ir(Me_4_Cp=CH_2_)(bpy)OH_2_]; (e) [Rh(Cp*)(en)OH]; (f) [Rh(Me_4_Cp=CH_2_)(en)OH_2_]; (g) superimposed structures of [Me_4_Cp=CH_2_]^2–^ dianion in optimized structure of [Rh(Me_4_Cp=CH_2_)(bpy)OH_2_] (black) and optimized structure of free dianion (blue).

These were performed to gain insight into the mechanism of deuteration and the difference in behavior between the bpy and phen complexes, and inactive Rh^III^en and Ir^III^bpy complexes. Recently it was shown that the DFT modeling can be very helpful to elucidate the mechanism of hydrogen formation catalyzed by RhCp* (bipy) involving the Cp* protonation.^38^ We explored a mechanism for H/D exchange involving metal–ligand-assisted (M–OH) deprotonation of a Cp* ring methyl to generate a transition state with a bound dianionic ligand [Me_4_Cp=CH_2_]^2–^ which could then rapidly gain a deuteron from solvent to generate a –CH_2_D substituent. Rapid methyl and ring rotation can lead to the sequential deuteration of all 15 methyl protons. We modeled the hydroxido adducts using the CAM-B3LYP functional with a CEP-31G basis set. In the cases of both Rh^III^ and Ir^III^ bpyridine complexes [M(Cp*^–^)(bpy)OH]^+^, the optimized structures show a relatively close M–O(H)···HCH_2_ contact between the bound hydroxide and a Cp* ring methyl (Fig. 2a), being slightly shorter for Rh (2.165 Å) than Ir (2.227 Å), (Fig. 2b, Table S4†). In contrast, for the en–Rh complex (**11**), the hydroxide is oriented so as to optimize H-bonding with an en NH_2_ proton, Rh–O(H)···HNH(en) 2.053 Å, Fig. 2e.

Next we determined the energies of the structures after transfer of a proton from a Cp* methyl group to coordinated hydroxide, giving coordinated dianionic [Me_4_Cp=CH_2_]^2–^ and H_2_O ligands, Fig. 2c and d. This state for Rh is only 35 kJ mol^–1^ higher in energy than the original R–OH/Cp*-state (Table S5, Fig. S10†, see the pdb files Rh_bipy_H_2_O.pdb and Rh_bipy_OH.pdb in ESI†). However, for the Ir complex it is 70 kJ mol^–1^ higher (Table S5†). The complementary results obtained with TPSSh functional and QZVP basis set were similar, revealing that the [Me_4_Cp=CH_2_]^2–^/H_2_O-coordinated chelates lay 28 kJ mol^–1^ for Rh and 62 kJ mol^–1^ for Ir above Cp*/M–OH. In the aqua adduct of the en Rh-complex, the H_2_O ligand is effectively outside the first coordination sphere (Rh–O 3.365 Å, Fig. 2f). In addition, the cyclopentadienyl-based dianion shifts “upwards”, with respect to the plane that approximately bisects that defined by the central ion and chelated nitrogens (Fig. 2f). The superimposed structures of both isomers of Rh and Ir bpy complexes, as well as the Rh en complex are shown in Fig. S11.† The energy barrier associated with the rotation of the cyclopentadienyl ring is low (calculated 6 kJ mol^–1^), which together with rapid methyl rotation, would allow sequential deprotonation and deuteration of each of the ring methyl protons.

Remarkably, the comparison of the geometries of (a) free deprotonated Cp* ligand (optimized with CAM-B3LYP/CEP-31G), (b) deprotonated Cp* in the optimized structures of the complex, and (c) that of the neutral fulvene Cp* derivative (optimized with CAM-B3LYP/CEP-31G) suggest that the latter two are close in energy to each other, while the former reveals an sp^3^ character of the CH_2_ carbon (Fig. S12 and S13†). This allows an alternative description of the complex containing deprotonated Cp* as [Rh^I^/Ir^I^(bpy)(H_2_O)(Me_4_Cp=CH_2_)]^+^. Thus, deprotonation of Cp* might introduce new pathways of activity including addition reactions of a bound fulvene, and redox reactions of rhodium.

Our calculations suggest that the π-acceptor strength of the *N*,*N*′-chelated ligand plays an important role in stabilizing the aqua/dianionic [Me_4_Cp=CH_2_]^2–^ intermediate in which H/D exchange can readily occur. Within the series of chelates of varying π-character of the *N*,*N*′ ligands, the stability of the intermediates increases with increasing π-acceptor character of the ligand. When we increased the π-acceptor strength by adding strongly electron-withdrawing groups as in 1,2-diimino-4,5-dicyanobenzene, the energy difference decreased to only 13 kJ mol^–1^ above the OH adduct (Table S5).† Interestingly, the energy difference for each Ir system is systematically 35–40 kJ mol^–1^ higher in energy than that for the corresponding Rh complexes (Table S5†). Thus, DFT modeling implies that the stability of the dianion of the intermediate (or formation of Rh^I^/Ir^I^ fulvene complex) is related to delocalization of the negative charge onto the *N*,*N*′-chelated ligand *via* the d-orbitals of the metal. This is facilitated by the π-acceptor character of the *N*,*N*′ ligand and is less effective for Ir than for Rh. Alternatively, the enhanced π-acceptor character of the *N*,*N*′-chelate stabilizes the lower Rh^I^ oxidation state.

### Reactions with GSH

Since ene–thiol reactions are well known, and glutathione (GSH, γ-l-Glu-l-Cys-Gly) is prevalent in cells at millimolar concentrations, reactions of GSH were studied with **1** under conditions in which activation of Cp* ring methyls was expected. A solution of **1** and excess GSH at pH 7 gave rise only to MS peaks assignable to [(η^5^-Cp*)Rh(bpy)(SG)]. Interestingly, the presence of GSH restricted, but did not prevent, ring methyl deuteration (up to 7 days incorporated at pH 7 after 3 days at 310 K, Fig. S14†), DFT calculations on interactions with GSH and modelling (a) [(Cp*)Rh(bpy)(GS)]/H_2_O, (b) [(Cp*)Rh(bpy)(OH)]^+^/GSH, (c) [Rh(Me_4_Cp=CH_2_)(bpy)(OH_2_)]^+^/GSH, and (d) [Rh(Me_4_Cp=CH_2_)(bpy)]^+^/GSH/H_2_O, revealed [Rh(bpy)Cp*(SG)] as the most stable complex (Fig. S15†) formed by coordination of deprotonated GS^–^ sulfur rather than an ene–thiol adduct. Interestingly, the adduct is further stabilized by specific interaction of a deprotonated GS carboxylate group with a Cp* methyl group and bpy 5-H and 6-H (2.50, 2.64 and 2.30 Å, respectively).

### Reaction with *N*-methylmaleimide

**Fig. 3:**
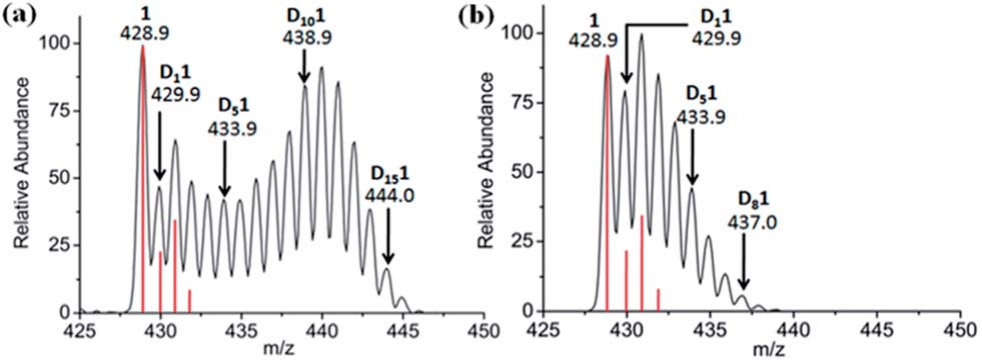
Effect of *N*-methylmaleimide on deuteration of [(η^5^-Cp*)Rh(bpy)Cl]^+^, **1**, in 60% MeOD-*d*_4_/40% D_2_O after 7 days at 310 K monitored by MS. (a) 2 mol equiv. and (b) 4 mol equiv. *N*-methylmaleimide. The red lines show the isotopic distribution for the non-deuterated complex.

Attempts were made to trap a Rh^I^–bound fulvene intermediate *via* [4+2] cyclo-addition between the dienophile, *N*-methylmaleimide, and complex **1**. Even after 5 days at 310 K no such adduct was observed by ^1^H NMR or MS, presumably due to steric crowding by the Cp* ring methyls hindering approach of the dienophile. However, *N*-methylmaleimide clearly interacted with complex **1**, [(η^5^-Cp*)Rh(bpy)Cl]PF_6_, since with **2** mol equiv. of *N*-methylmaleimide present in 60% MeOD-*d*_4_/40% D_2_O, complete deuteration was not observed even after 7 days, rather MS peaks for all D_1_ to D_15_ sequential steps (Fig. 3a) were present. With 4 mol equiv. of *N*-methylmaleimide, only D_1_–D_9_ species were observed after 7 days (Fig. 3b). A DFT study suggested that *N*-methylmaleimide interacts strongly with the coordinated water in the fulvene intermediate through a short maleimide C=O···H–OH bond of 1.68 Å rather than attacking the fulvene double bonds (Fig. S16†). The difference in energy of the complex–maleimide pair and the sum of the energies of the isolated complex and maleimide is 50 kJ mol^–1^. Thus the fulvene is too crowded to allow a strong interaction with the dienophile, but the strong interaction of *N*-methylmaleimide with coordinated H_2_O may result in lower acidity, thus explaining the lack of deuteration.

### Trapping the Rh^I^–fulvene intermediates

[4+2] Diels–Alder reactions with the exocyclic double bond of fulvene as dienophile have been reported in the literature.^30^,^31^ The conjugated dienes, isoprene and (9*Z*,11*E*)-linoleic acid, were used in attempts to trap the possible fulvene intermediate as [4+2] cyclo-addition Diels–Alder adducts. These are both biologically-important, natural dienes. Isoprene is the most abundant hydrocarbon in human breath, with an estimated human production of 17 mg per day.^32^,^33^ (9*Z*,11*E*)-Linoleic acid is a common dietary conjugated fatty acid of much interest because of its potential health effects.^34^,^35^

**Fig. 4:**
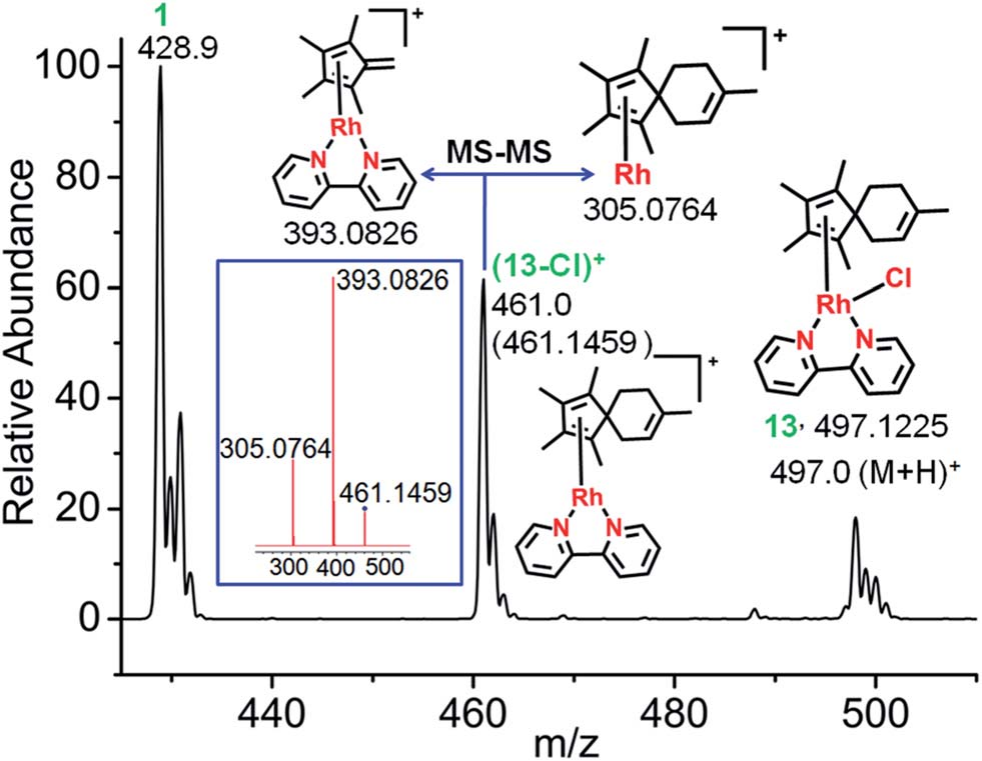
Characterization of the Diels–Alder adduct of complex **1** with isoprene (complex **13**) by ESI-MS and MS-MS. The mass spectrum shows the formation of the fulvene-isoprene [4+2] cyclo-addition adduct with coordination of η^4^-1,2,3,4,8-pentamethylspiro[4,5]deca-1,3,7-triene to Rh^I^, and its fragmentation by MS-MS (blue box).

First, the reaction between complex **1** in 60% MeOD-*d*_4_/40% D_2_O at 310 K and isoprene was studied, since isoprene was expected to offer minimal steric hindrance for the reaction with the proposed exocyclicfulvene C=CH_2_. The ESI-MS spectra clearly indicated formation of a [4+2] cyclo-addition adduct, with the peaks at *m*/*z* = 497.1225 assignable to the Rh^I^ complex (13+H)^+^ (Fig. 4). HR-MS data confirmed the formulations C_25_H_30_ClN_2_Rh, Table S6.† Therefore, the data provide strong evidence for the formation of a fulvene intermediate during Cp*–CH_3_ activation. As expected, adduct formation hinders deuteration. ESI-MS data suggested that the Rh^I^–Cl bond in the fulvene adduct is weak, giving rise to the peak at 461.1459 assignable to [**13**-Cl]^+^. This is consistent with the report of a very long Rh^I^–Cl bond (*ca.* 2.55 Å) in a Rh^I^–(Cp*H) complex.^27^ DFT calculations indicated lengthening of the Rh–Cl bond by 0.2 Å in the Rh^I^ system compared to Rh^III^, making a square-planar Rh^I^ (4d^8^) adduct accessible (Fig. S17†).

Adduct formation was also evident from LC-MS data (Fig. S18†), showing a reverse-phase HPLC peak at 25.6 min with an *m*/*z* of 461.16 assignable to [**13**-Cl]^+^, as was also observed by ESI-MS.

**Fig. 5:**
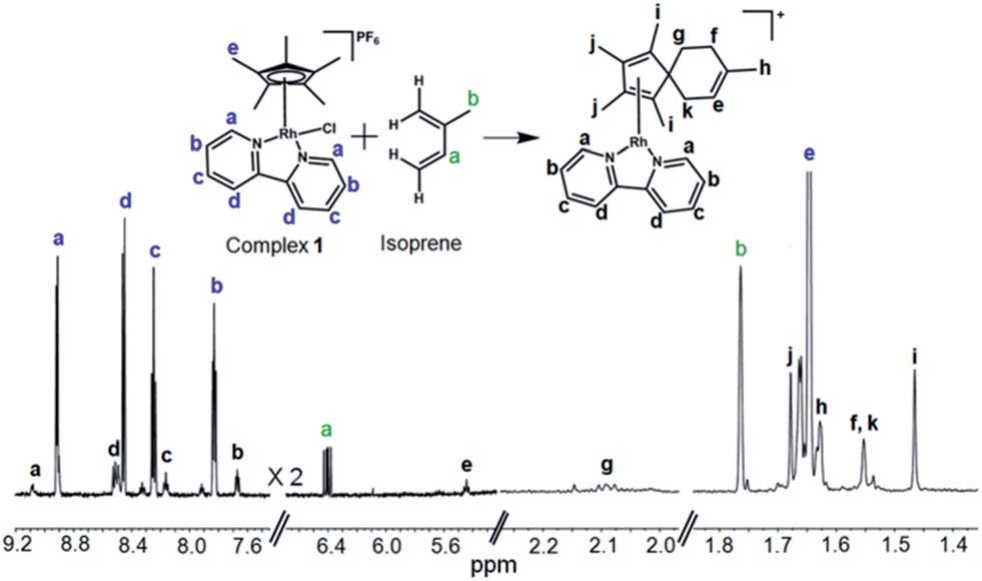
600 MHz ^1^H NMR characterization of the isoprene adduct complex **13** in 60% MeOD-*d*_4_/40% D_2_O after 3 days at 310 K. *δ*_H_ 9.09 (d, 2H, *J* = 6.0 Hz), 8.51 (d, 2H, *J* = 6.1 Hz), 8.17 (t, 2H, *J* = 8.0 Hz), 7.67 (dd, 2H, *J*_1_ = 8.1 Hz, *J*_2_ = 6.2 Hz), 5.44 (t, 1H, *J* = 8.6 Hz), 2.10 (t, 2H, *J* = 7.0 Hz), 1.68 (s, 6H), 1.63 (s, 3H), 1.55 (s, 4H), 1.47 (s, 6H). The peaks form excess isoprene (10 mol equiv.) from 4.9–5.2 ppm are shown in Fig. S19.†. 2D COSY NMR data (Fig. S20†) indicated the coupling of peak “f” (1.55 ppm) with peak “g” (2.10 ppm), and peak “k” (1.55 ppm) with “e” (5.43 ppm). The 2D NOESY spectrum (Fig. S21†) indicated cross-correlation of peak “g” (2.10 ppm) with peaks “h” (1.47 ppm) and “k” (1.55 ppm) suggesting that these protons are closer than *ca.* 5 Å (for a model see Fig. S21†).

The formation such a [4+2] cyclo-addition adduct was further confirmed by ^1^H NMR studies. Time-dependent 600 MHz ^1^H NMR spectra of the reaction mixture of complex **1** and isoprene (10 mol equiv.) in 60% MeOD-*d*_4_/40% D_2_O showed a decrease in the intensity of the isoprene peaks with time, Fig. S19,† accompanied by an increase in the intensity of peaks arising from the formation of the adduct (complex **13**). The new peaks due to formation of the [4+2] cyclo-addition adduct are assigned in Fig. 5. The assignments to a Diels–Alder cyclo-addition adduct were made on the basis of ^1^H COSY and NOESY data (Fig. S20 and S21†).

The MS and NMR data provide the first experimental evidence for the formation of a fulvene intermediate trapped during the course of Cp*–CH_3_ activation under such mild conditions in aqueous media, and this appears to be the first report of a Diels–Alder reaction of a metal-bound fulvene in aqueous solution. There appears to be only one previous report on the promotion of a Diels–Alder reaction through enhancement of diene reactivity through metal coordination.^39^ Interestingly, we detected the same Diels–Alder adduct by HRMS when complex **1** was incubated with isoprene (10 mol equiv.) in RPMI-1640 cell culture medium supplemented with 10% fetal calf serum, a typical medium used for culture of cancer cells (Fig. S22†).

**Fig. 6:**
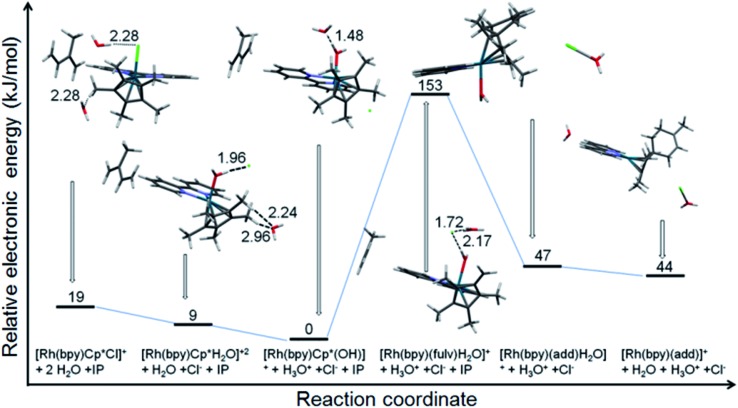
Electronic energy profile for the reaction of [(Cp*)Rh(bpy)Cl]^+^ with isoprene in water at neutral pH (calculated using CAM-B3LYP/CEP-31g, solvent = water, IEFPCM model). The pdb files (pdb files bp1-6.pdb) of the optimised structures of all six species are supplied as ESI.†

The reaction of [(Cp*)Rh(bpy)Cl]^+^ with isoprene in water was modeled using DFT calculations (Fig. 6). The results indicate the relative stability of the initial Rh^III^ complex with the lowest energy corresponding to [(Cp*)Rh(bpy)OH]^+^. The formation of the Diels–Alder adduct from the fulvene species [(Me_4_Cp=CH_2_)Rh^I^(bpy)OH_2_]^+^ is energetically very favourable (106 kJ mol^–1^), while the corresponding end product [(adduct)Rh^I^(bpy)]^+^ is presumably driven by entropic effects, with a calculated energy difference between it and the [(adduct)Rh^III^(bpy)](OH_2_) of only 3 kJ mol^–1^. The change of the oxidation state is suggested by the calculated Rh–N bond lengths of 2.141 and 2.192 Å for the species with coordinated water, and 2.106 and 2.102 Å for the final product.

**Fig. 7:**
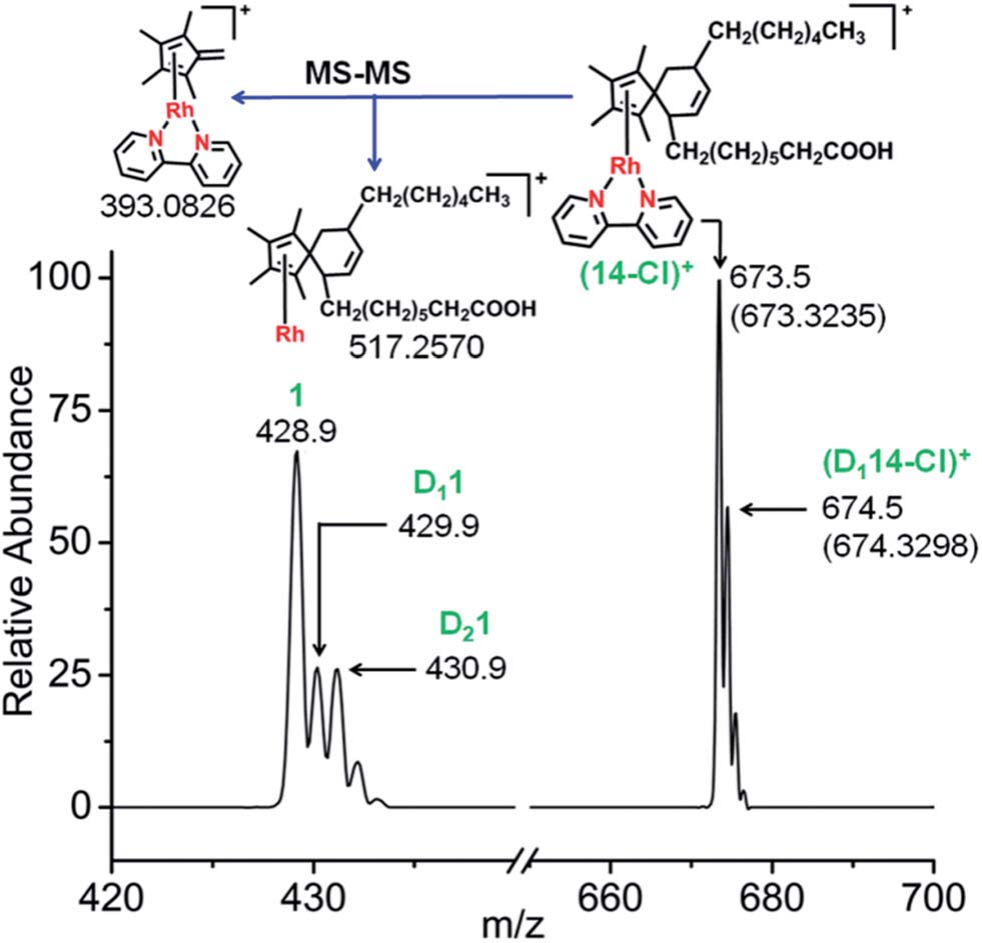
Mass spectra of complex **1** after reaction with conjugated fatty acid, (9*Z*,11*E*)-linoleic acid showing the formation of the fulvene-linoleic acid [4+2] cyclo-addition adduct, complex **14**.

Next, the reactions of complex **1** with the conjugated fatty acid, (9*Z*,11*E*)-linoleic acid, under similar experimental conditions in water, and in RPMI-1640 cell culture medium supplemented with 10% of fetal calf serum, were studied. The HRMS and MS-MS data again clearly indicated the formation of the fulvene-diene cyclo-addition adduct (Fig. 7 and S23†), with peaks at *m*/*z* = 673.3235 and 674.3298 assignable to the Rh^I^ complexes [**14**-Cl]^+^ and [D_1_**14**-Cl]^+^, respectively. HR-MS data confirmed the formulations as C_38_H_54_N_2_O_2_Rh and C_38_H_53_DN_2_O_2_Rh, Table S7.† Interestingly, adduct formation is reversible, as shown in Fig. S24.† Dilution of the reaction mixture leads to the regeneration of the parent Rh^III^ complex suggesting possible use as a drug delivery vehicle.

We investigated the formation of [4+2] cyclo-addition adducts between the biphenyl-substituted Cp* complex **10**, an active anticancer complex, with isoprene in 60% MeOD-*d*_4_/40% D_2_O at 310 K. This gave rise to a product complex **15**, as evidenced by ESI-MS, HRMS and LC-MS data (Fig. S25 and S26†), which suggested that a similar cyclo-addition adduct was formed. The peaks at *m*/*z* = 623.1928, 624.1980 and 625.2030 are assignable to the Rh^I^ complexes [**15**-Cl]^+^, [D_1_**15**-Cl]^+^ and [D_2_**15**-Cl]^+^ respectively (Fig. S25†). HR-MS data confirmed the formulations C_38_H_36_N_2_Rh, C_38_H_35_DN_2_Rh and C_38_H_34_D_2_N_2_Rh. The presence of isomers in the product was evident from LC-MS data (Fig. S26†), and reflect the non-equivalence of methyl deprotonation sites on the bound Cp^*x*PhPh^. Two reverse-phase HPLC peaks were detected at 32.3 min with an *m*/*z* of 623.49 assignable to [**15**-Cl]^+^ and 35.2 min with an *m*/*z* 623.48. These data are in good agreement with the observed ESI-MS results.

They, therefore, establish the formation of Rh^I^–fulvene intermediates during Cp*–CH_3_ activation in reactions with conjugated dienes which are abundant in the body. These observations are of particular interest for the future design of organometallic half-sandwich anticancer complexes.^13^ For example, it might be possible to use such conjugates to deliver and release complexes in specific cellular organelles.

## Conclusions

**Fig. 8:**
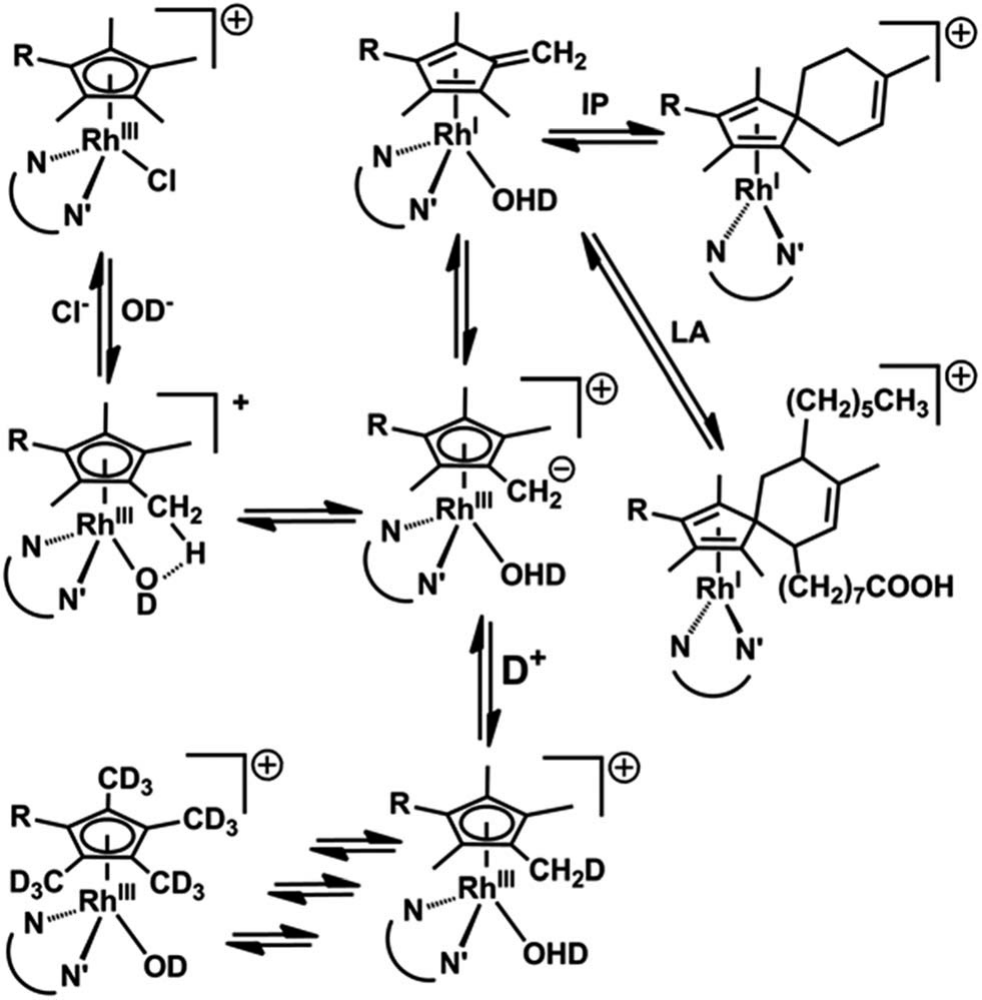
Reaction pathways for Cp^*x*^ methyl deuteration and Rh^I^ fulvene-diene adduct formation.

It is difficult to activate C–H bonds of hydrocarbons, especially CH_3_ groups, in water under mild conditions, but we show that this is facile for certain Rh^III^ cyclopentadienyl complexes. DFT calculations revealed the role of strategically-placed Rh-OD groups in abstracting protons from coordinated Cp^*x*^ rings aided by the presence of chelated π-acceptor diamine ligands such as bpy or phen (Fig. 8). These ligands help to stabilize the deprotonated Cp^*x*^–H dianion. The Cp^*x*^ ring behaves like a ‘molecular twister’ with low barriers to rotation about the Cp^*x*^–Rh and ring-CH_3_ axes, allowing rapid sequential deuteration. The readily prepared deuterated products are useful for ^2^H NMR studies in situations where overlap in ^1^H NMR spectra hinders studies, for example in cell culture media (Fig. S9†).

The DFT calculations predicted the accessibility of Rh^I^–fulvene intermediates formed *via* 2-electron transfer from the dianionic Cp^*x*2–^ ligand. We were not able to generate addition products between the thiol of glutathione and the exocyclic C=CH_2_, or with the dienophile *N*-methylmaleimide, although they can control the extent of ring deuteration (Fig. 3 and S14†). However, the trapping of the Rh^I^–fulvene intermediates was surprisingly facile in Diels–Alder [4+2]-cycloaddition reactions with natural conjugated dienes. This appears to be the first report of metal-assisted *in situ* formation of a dieneophile, and trapping of that dieneophile by a Diels–Alder cyclo-addition reaction of a metal-bound fulvene in aqueous solution.

Adducts with isoprene, an abundant diene in human breath, and with the common dietary diene conjugated (9*Z*,11*E*)-linoleic acid formed readily in aqueous solution. They might easily form in cells, and influence the redox state of cells and biological activity of this class of organo–rhodium anticancer complexes. The Diels–Alder adducts formed readily in biological media, *e.g.* cell culture medium (Fig. S22 and S23†) and since they are reversible (Fig. S24†), open up the possibility of designing novel drug targeting and delivery systems. Interestingly, the most active anticancer complexes in the limited set we have studied here, are those that have activated Cp^*x*^ rings (Table S3†). The possible involvement of such Diels–Alder adducts in the biological activity of this class of complexes will be studied in future work.

## Conflicts of interest

There are no conflicts to declare.

## Supplementary Material

Supplementary information

Crystal structure data
