# Supplementary material for: New activation mechanism for half-sandwich organometallic anticancer complexes
Source: Chem Sci. 2018 Mar 1;9(12):3177–85. doi: 10.1039/c7sc05058e (PMC5916112; doi:10.1039/c7sc05058e)
Supplement: Supplementary file 1 [file SC-009-C7SC05058E-s001.pdf]

Electronic Supplementary Material (ESI) for Chemical Science.
This journal is © The Royal Society of Chemistry 2018

Electronic Supplementary Information (ESI)

New Activation Mechanism for Half-sandwich Organometallic Anticancer Complexes

Contents

S.1 Materials

S.2 NMR Spectroscopy

S.3 Electrospray ionization mass spectrometry (ESI-MS)

S.4 nESI-FT-ICR mass spectrometry

S.5 X-ray crystal structures.

S.6 pH measurements

S.7 Elemental analysis

S.8 DFT calculations: hydride mechanism; effect of solvent; kinetic barriers; M(III) versus M(I) intermediates; interaction of complex **1** with GSH

S.9 Synthesis and characterization data for complexes **1-12**

S.10 Aqueous solution chemistry

S.10.1 Hydrolysis and pK_a determinations

S.10.2 Cyclopentadienyl deuteration by ^{1}H NMR spectroscopy

S.10.3 Kinetic experiments for deuteration of cyclopentadienyl ligands

S.10.4 Effect of complex hydrolysis on deuteration of cyclopentadienyl ligands

S.10.5 Effect of methanol on deuteration of cyclopentadienyl ligands

S.10.6 Effect of pH on deuteration of cyclopentadienyl ligands

S.10.7 Reversibility of H/D exchange reactions

S.11 Reaction of complex **1** with N-methylmaleimide

S.12 Reaction of complex **1** with isoprene: formation of complex **13**

S.13 Reaction of complex **1** with conjugated (9Z,11E)-linoleic acid: formation of complex **14**

S.14 Reaction of complex **10** with isoprene: formation of complex **15**

S.15 ^{2}H NMR of $[(D_{15}\text{-}\eta^5\text{-Cp*})Rh(bpy)Cl]PF_6$

Tables S1, S2 Crystallographic data

Table S3 IC_{50}, pK_a and hydrolysis data

Tables S4, S5 DFT Computational data

Tables S6, S7 HRMS and MS-MS data for complexes **13** and **14**

Figure S1 X-ray crystal structures of complexes **1**, **3**, **4**, **6**, **7**, **9**, **10** and **12**

Figures S2-S8 NMR/MS studies of deuteration

Figure S9 Examples of ^{2}H NMR studies

Figure S10 Energy profile for the deprotonation of CH_3 of Cp*

Figure S11 Optimized structures of M(Cp^x)(*N,N'*)OH and M([Me_4Cp=CH_2)(N,N')OH_2

Figure S12 Optimized structures of [Cp*=CH_2]$^{2-}$, coordinated Cp*=CH_2 fragment, and optimized structure of the neutral fulvene Cp*=CH_2

Figure S13 Kohn-Sham orbitals for [(Cp*)Rh(bpy)(OH)]$^+$, and [Rh(bpy)(H_2O)Cp*=CH_2]$^+$

Figure S14 ESI-MS of [(η^5-Cp*)Rh(bpy)(SG)]

Figure S15 Optimized structures and relative energies (kJ/mol) of glutathione complexes and adducts of {(Cp*)Rh(bpy)}

Figure S16 Optimised structure of the adduct of N-methyl maleimide and fulvene intermediate [Rh(bpy)(H_2O)(Me_4Cp=CH_2)]$^+$.

Figure S17 Energy profile diagram of Rh^I-fulvene-isoprene adduct formation

Figures S18-S23 Characterization of Rh^I-fulvene Diels-Alder adducts with isoprene and conjugated (9Z,11E)-linoleic acid by MS and 1D and 2D NMR

Figure S24 Reversibility of formation of complex **14**, the Rh^I-fulvene--conjugated (9Z,11E)-linoleic acid adduct

Figures S25, S26 Characterization of Rh^I-fulvene Diels-Alder adducts of isoprene and complex **10** by ESI-MS, HRMS and LC-MS

References

S.1. Materials

Rhodium(III) trichloride hydrate was purchased from Precious Metals Online (PMO Pty Ltd.) and used as received. Ethylenendiamine (en) was purchased from Sigma-Aldrich and freshly distilled prior to use. The ligands 3-phenyl-1,2,4,5-tetramethyl-1,3-cyclopentandienyl (Cp^{xPh}) and 3-biphenyl-1,2,4,5-tetramethyl-1,3-cyclopentandienyl (Cp^{xPhPh}) were synthesized following the methods in the literature.[1,2] 2,2'-Bpyridine, 4,4'-dimethyl-2,2'-dipyridine, 1,10-phenanthroline, 4-bromo-biphenyl, 4-bromo-biphenyl, *n*-butyllithium in hexane 1.6 M, phenyllithium in ether (1.8 mM), 2,4-pentamethylcyclopentadiene, 2,3,4,5-tetramethyl-2-cyclopentanone, isoprene, N-methylmaleimide, conjugated (9Z,11E)-Linoleic acid and reduced glutathione were obtained from Sigma-Aldrich. Magnesium sulphate, ammonium hexafluorophosphate, silver nitrate, sodium chloride, hydrochloric acid were obtained from Fisher Scientific. DMSO-d_6, MeOD-d_4, D_2O, acetone-d_6, NaOD solution (40 wt% in D_2O, 99+ atom% D) and DNO_3 solution (65 wt% in D_2O, 99 atom% D) for NMR spectroscopy were purchased from Sigma-Aldrich and Cambridge Isotope Labs Inc. Non-dried solvents used in synthesis were obtained from Fisher Scientific and Prolabo.

S.2. NMR Spectroscopy

^{1}H NMR spectra were acquired in 5 mm NMR tubes at 298 K or 310 K on either Bruker AV-400 or Bruker AV III 600 spectrometers. Data processing was carried out using XWIN-NMR version 3.6 (Bruker U.K. Ltd). ^{1}H NMR chemical shifts were internally referenced to TMS *via* 1,4-dioxane in D_2O (δ = 3.66 ppm). 1D spectra were recorded using standard pulse sequences. Typically, data were acquired with 16, 32 or 128 transients into 32 k data points over a spectral width of 20 ppm, and for the kinetic experiments, 32 transients into 32 k data points over a spectral width of 20 ppm using a relaxation delay of 2 s.

COSY and NOESY spectra were acquired in 5 mm NMR tubes at 298 K on Bruker AVIII-600 spectrometer at 600.13 MHz with 1,4-dioxane in D_2O (δ = 3.66 ppm) as internal standard. Spectra were recorded using a standard pulse sequence (2D homonuclear correlation via dipolar coupling with gradient pulses in the mixing time). Typically, data were acquired with 16 accumulations and 2048 × 256 sampling data points with a NOESY mixing time of 0.6 s. Data processing was carried out using XWIN-NMR version 3.2 (Bruker U.K. Ltd).

^{2}H NMR spectra were acquired in 5 mm NMR tubes at 298 K on a Bruker AV-500 spectrometer at 76.77 MHz using standard pulse sequence for ^{2}H lock switch unit. Typically, data were acquired with 64 scans into 16k data points over a spectral width of 20 ppm using a relaxation delay of 0.1 s. Data processing was carried out using XWIN-NMR version 3.6 (Bruker U.K. Ltd).

S.3. Electrospray ionization mass spectrometry (ESI-MS)

Positive ion electrospray mass spectra were obtained on a Bruker Daltonics Esquire 2000 ion trap mass spectrometer. All samples were prepared in methanol (100%). Data were processed using Data-Analysis version 3.3 (Bruker Daltonics).

HR-MS analysis was carried with a Bruker MaXis plus Q-TOF mass spectrometer equipped with electrospray ionisation source. The mass spectrometer was operated in electrospray positive ion mode with a scan range 50-2,400 m/z. Source conditions were: end plate offset at -500 V; capillary at -4000 V; nebulizer gas (N_2) at 0.5 bar; dry gas (N_2) at 4 L/min; dry temperature at 453 K. Ion tranfer conditions as: ion funnel RF at 200 Vpp; multiple RF at 200 Vpp; quadruple low mass set at 50 m/z; collision energy at 5.0 eV for MS and 10-20 eV for MS/MS, MS/MS isolation window 10; collision RF at 500-2000 Vpp; transfer time set at 50-150 µs; pre-pulse storage time set at 5 µs. Calibration was carried out with sodium formate (10 mM) before analysis.

S.4. nESI-FT-ICR mass spectrometry

Samples were analyzed *via* nano-electrospray ionization (nESI)-Fourier Transform Ion Cyclotron Resonance Mass Spectrometry (FT-ICR MS). All experiments were carried out on a Bruker SolariX FT-ICR Mass Spectrometer, fitted with a 12 tesla actively shielded magnet (Bruker Daltonik GmbH, Bremen, Germany). Samples were diluted 1000-fold from NMR samples/reaction mixtures with appropriate deuterated/non-deuterated solvents (to ~1µM), an aliquot of each sample (10-20 µL) was ionized from ~1 µm glass nESI capillaries using a capillary voltage of 900-1200V, ions were accumulated for 0.01 s in the hexapole-based collision cell before transfer to the ICR cell for detection. Ions of m/z 147-3000 were excited using a frequency sweep excitation and detected for 3.2 s transient length (8M (16-bit) data points), producing a resolving power of ~850,000 (at 400 m/z) for all spectra. Mass spectra were externally calibrated using a quadratic calibration function/internally using single point calibrations and then manually interpreted and assigned via Data Analysis v4.2 (Bruker Daltonik GmbH, Bremen, Germany).

S.5. X-ray crystal structures

Diffraction data were collected on an Oxford Diffraction Gemini four-circle system with a Ruby CCD area detector. All structures were refined by full-matrix least squares against F^2 using SHELXL 97[3] and were solved by direct methods using SHELXS[4] (TREF) with additional light atoms found by Fourier methods. Hydrogen atoms were added at calculated positions and refined using a riding model. Anisotropic displacement parameters were used for all non-H atoms; H-atoms were given an isotropic displacement parameter equal to 1.2 (or 1.5 for methyl) times the equivalent isotropic displacement parameter of the atom to which they are attached. The data were processed by the modelling program Mercury 1.4.1. The Rh-Cl distances are in the range 2.385-2.403 Å, with complexes containing extended Cp^x rings showing slightly longer bond lengths. The Rh-Cp^x centroid distance of 1.778-1.795 Å shows little change with extension of the Cp* ring. Interestingly, complexes **6**, **7** and **9** have one Rh-N distance slightly longer than the other. For complex **12**, a suitable crystal was selected and mounted on a glass fibre with Fromblin oil and placed on an Xcalibur Gemini diffractometer with a Ruby CCD area detector. The crystal was kept at 150(2) K during data collection. Using Olex2,[5] the structure was solved with the ShelXT[6] structure solution program using Intrinsic Phasing and refined with the ShelXL[7] refinement package using Least Squares minimization. The asymmetric unit contains the Rh complex, two PF_6 counter ions and a partially occupied water molecule. No hydrogens were located on the water molecule. The complex was modeled as disordered over two closely related positions. The occupancy was originally linked to a free variable that settled around 85:15 and the occupancy of the components was fixed at this value for the rest of the refinement. The PF_6 counter ion (P20_F26) was modeled as disordered over two closely related positions again linked to 85:15 occupancy. Additionally, the major component was also modeled as disordered where the components were related by a rotation about the meridian. The occupancy of these components (F23-F26: F23A-F26A) was linked to a free variable which refined to 76:24 (of

the 85% occupancy). Minor components were refined isotropically and several DFIX, DANG and SIMU restraints were used to give the minor components reasonable bond lengths, angles and thermal parameters.

X-ray crystallographic data for complexes **1**, **3**, **4**, **6**, **7**, **9**, **10** and **12** (all as PF_6salts) are available as Supporting Information and have been deposited in the Cambridge Crystallographic Data Centre (CCDC reference numbers 1420046-1420052 and 1569283, respectively).

S.6. pH* measurements.

pH* values (pH meter reading without correction for the effect of deuterium) were determined at ambient temperature using a minilab IQ125 pH meter equipped with a ISFET silicon chip pH sensor and referenced in KCl gel. The electrode was calibrated with Aldrich buffer solutions of pH 4, 7 and 10. pH* values were adjusted with NaOD or DNO_3 solutions in D_2O.

S.7. Elemental analysis.

Elemental analyses were performed by Warwick Analytical Service using an Exeter Analytical elemental analyzer (CE440).

S.8. DFT calculations

The electronic energies for all complexes under study were calculated with Gaussian 09, version D.[8] using CAM-B3LYP functional[9] and CEP-31G basis set.[10-12] For comparison, the Ir and Rh complexes were modeled with the TPSSh functional[13] and QZVP basis set[14,15] for both M(Cp^{*-})(bpy)OH and M($[Me_4Cp{=}CH_2]^{2-}$)(bpy)H_2O isomers. In each case the structure was optimized using the standard optimization procedure of Gaussian 09, followed by the frequency calculations in order to find whether the true minimum was found. Ultrafine grid of integration was applied in all cases. In the case of M($[Me_4Cp{=}CH_2]^{2-}$)(bpy)H_2O isomers

calculated with TPSSh/QZVP method, the calculation of forces (Gaussian keyword CALCF) was applied. The proton transfer transition state and cyclopentadienyl anion rotation barrier for [Rh(Cp^-)(bpy)OH] was calculated using the QST3 method, followed by frequency calculations.

Hydride mechanism

A mechanism for H/D exchange that involves formation of a hydride intermediate was also considered. Such a mechanism operates for Cp^*Ir^{III} catalyzed H/D exchange of aromatic CH bonds in benzene derivatives in solvents such as MeOD-d_4.[16] H_2 was found to remain coordinated to Ir and is rather loosely bound to Rh. In both cases the H_2 isomer is greatly destabilized relative to the hydride complex (by 190 kJ mol^{-1} and by 120 kJ mol^{-1} for Ir and Rh, respectively). If it is assumed that the deuteration of Cp* in D-hydride complex proceeds *via* formation of coordinated HD, then this intermediate state presents a very high-activation barrier. Therefore this mechanism did not seem to be competitive with the Rh-OH mechanism.

Effect of solvent

Optimization calculations were also performed using methanol as solvent with the polarisable continuum model (with IEFPCM keyword of Gaussian). Frequency calculations confirmed that true minima were identified. The sequence of the energy differences for the hydroxide/aqua pairs Rh(bpy)Cp*OH/H_2O, Ir(bpy)Cp*OH/H_2O and Rh(en)Cp*OH/H_2O pairs is qualitatively the same as in the absence of methanol. The energy differences are in the Table below.

Pair	ΔE / kJ/mol<br>No solvent	ΔE / kJ/mol<br>MeOH model
Rh(bpy)Cp*OH/H_2O	35	49
Ir(bpy)Cp*OH/H_2O	70	83
Rh(en)Cp*OH/H_2O	60	70

Thus the incorporation solvent in DFT modelling adds ca.10-15 kJ/mol to the energy difference.

Kinetic barriers

The intermediate state of the transition between [Rh/Ir(bpy)Cp*OH] and [Rh/Ir(bpy)Cp*H_2O]$^{2-}$ was calculated. The saddle points are shown in Figure S10, the structures of the saddle points are in pdb files (Rh/Ir_qst3_solv). Frequency calculations for the transition states revealed one negative frequency, confirming its saddle point character.

This intermediate states would be relevant if we assume the following mechanism for the H-D exchange: the [Rh/Ir(bpy)Cp*H_2O] reacts with CD_3OD to give [Rh/Ir(bpy)(Cp^{-2}*)HDO], which relaxes back to give [Rh/Ir(bpy)(CpD*)OH] and [Rh/Ir(bpy)(Cp*)OD]. Interestingly, the intermediate state lies closer to the [Ir(bpy)(Cp*)H_2O]$^{2-}$ (activation barrier of 13 kJ/mol) than to the Rh analogue (26 kJ/mol). Thus, the Cp*$^{2-}$/OH complex is not only thermodynamically, but also kinetically more stable for Rh than for Ir. For comparsion of the superimposed structures see Fig. S11.

Interestingly, a mechanism for a related reaction of [Rh($PTA)_2$(Cp*)Cl] (PTA = 7-phospha-1,3,5-triazaadamantane), proposed on the basis of DFT modelling involves a similar

transition state with deprotonated methyl group and formation of an aqua complex.[17] The value of $\Delta G^{\neq}$ of 40 kJ/mol was obtained for the proton transfer step. Assuming negligible entropic effects, this value is close to that of $\Delta H^{\neq}$ of 59 kJ/mol calculated here for the isolated molecule.

A second plausible mechanism for D transfer involves direct transfer of D from CD_3OD to the $=CH_2$ carbon of Cp^{-2}*. The structure for a methanol molecule interacting with the OH group of [Rh(bpy)(Cp*)(OH)] can be considered a "ground state". A second structure is shown which was obtained after assuming that the starting structure contains [Rh(bpy)(Cp*)H_2O] and methoxide, i.e. Rh is still bound to H_2O but the Cp^{-2}* has abstracted a proton from methanol.

This is an interesting structure (pdb files Rh_bpy_OH_MeOH and Rh_bpy_H_2O_MeOH), a possible active state which may revert to the initial complex, with methanol exchanging the proton. Interestingly, the second species lie 54 kJ/mol higher in energy than the first, while the previously quoted difference for solvent-modelled [Rh(bpy)(Cp*)OH/H_2O] pair of 49 kJ/mol is very similar. These data show that the inclusion of solvent has little effect on the general conclusions discussed in the main text.

M(III) versus M(I) intermediates

Comparison of the geometries of a) free deprotonated Cp* ligand (optimized with CAM-B3LYP/CEP-31G), b) deprotonated Cp* in the optimized structures of the complex and c) that of the neutral fulvene Cp* derivative (optimized with CAM-B3LYP/CEP-31G, Fig S12) suggest that the two latter are close to each other, while the former reveals the sp^3 character of the CH_2 carbon. This is seen when comparing the C-CH_2 bonds in the optimized structures, which are 1.370 Å for the deprotonated Cp^* ligand in [Rh($Me_4Cp=CH_2$)(bpy)OH_2] and 1.367 Å in the optimized fulvene derivative of Cp* (neutral $C_5(CH_3)_4CH_2$). On the other hand in the optimized structure of free $[Me_4Cp=CH_2]^{2-}$ dianion this bond length

is 1.499 Å, thus corresponding to a C-C single bond. Moreover, for the former two structures, the C-CH_2 is planar, while for the latter it reveals the tetrahedral character of the CH_2 carbon, with a H-C-H angle of 110° (see the superimposed structures in Figure 2g in the main text).

Hence an alternative and more common description of the deprotonated M(III)(Cp^{*2-}) fragment would be as M(I)(fulvene), i.e., with a neutral C_5Me_4=CH_2 tetramethylfulvene ligand. Apart from the above structural features the observed stabilization of the deprotonated species of $[M(Cp^*=CH_2)(bpy)(H_2O)]^+$ by the strong π–acceptor ligand supports a formulation with a Rh(I) oxidation state. We discuss further below the merits of these descriptions.

Firstly we inspected the d-orbitals of $[Rh(bpy)(H_2O)Cp^*=CH_2]^+$ and its $[Rh(bpy)(OH)Cp^*]^+$ precursor. In Figure S13 the occupied and virtual molecular orbitals of pronounced d-character are shown for both systems. It is to be noted that: (i) the contour and arrangement on the energy scale is nearly identical; (ii) for both species there are two virtual MO's with d-character, revealing the shape of d_{z2} and d_{x2-y2} - like orbitals. Rh^{III} is a d^6 system, and Rh^I d^8, so in the latter case there should be only one unoccupied d-orbital. Moreover the similarity of the MO's for both system implies the same electronic structure for both of them. The other point to note in this regard is that the Rh-N(bpy) bond lengths do not differ much in the optimized structures of OH and H_2O complexes, whereas they might be expected to be longer for Rh(I). Finally, the calculated Mulliken charges for Rh are -1.07 in $[Rh(bpy)(OH)Cp^*]^+$ and -0.84 for $[Rh(bpy)(H_2O)Cp^*=CH_2]^+$, the opposite of what might be expected on going from Rh^{III} to Rh^I.

On the other hand, it is to be noted that Ciancaleoni *et al* proposed that the structure with fulvene corresponds to a square-planar Rh^I complex with weakly coordinated water (Rh-O distance of 2.49 Å).[17] Our results reveal that the elongation of the Rh-OH_2 bond is the

result of lowering of the π-acceptor properties of the nitrogen ligands. This distance changes from 2.28 Å for *N,N'*-ethylenediimine, 2.30 Å for 2,2'-bpyridine, 2.40 Å for 2-picolylamine up to 3.39 Å for ethylenediamine, the latter being inactive as far as the deuteration is concerned. The relative stability of these systems regarding the "ground state" with coordinated OH^- ligands correlates with shorter water-rhodium bonds. Thus, the systems preferring the four coordination show no activity in deuteration of the Cp* ligand.

Summarizing, the species active in the catalytic deuteration have the characteristics of both Rh^{III} and Rh^{I} complexes. However, since we have used DFT calculations there is no implicit assignment of formal charges to the metal and the ligands.

Interaction of complex 1 with GSH

DFT calculations on the interaction of GSH with the $\{Rh(bpy)Cp^*\}^{2+}$ and its derivatives were performed using CAM-B3LYP/CEP-31, the Polarizable Continuum Model (PCM) with the integral equation formalism variant (IEFPCM), and water as solvent. Four different species were modeled: a) [(Cp*)Rh(bpy)GS]/H_2O with deprotonated glycine carboxylate and zwitterionic glutamine; b) $[(Cp^*)Rh(bpy)OH]^+$/GSH with deprotonated glycine carboxylate and zwitterionic glutamine; c) $[Rh([Me_4Cp{=}CH_2)(bpy)OH_2]^+$/GSH; d) $[Rh([Me_4Cp{=}CH_2)(bpy)]^+$/GSH/$H_2O$. In each case the starting structure of glutathione contained the zwitterionic glutamine and deprotonated carboxyl group of glycine. The results are depicted in Figure S15. The most stable complex is formed by coordination of deprotonated GS^- sulfur. The [(Cp*)Rh(bpy)(SG)] species is additionally stabilized by specific interaction of a deprotonated carboxylate group with a Cp* methyl group and bpy 5-H and 6-H, with the distances of 2.50, 2.64 and 2.30 Å, respectively. Any attempt to model the complex with coordinated protonated –SH group led to a dissociation of GSH. The substitution of GS by an hydroxyl group, i.e. to give $[(Cp^*)Rh(bpy)OH]^+$/GSH species, is

165 kJ/mol higher in energy yet, interestingly, the specific interaction of GSH with the complex is retained, with the glycine carboxyl oxygen displaying short (2.04, 2.10 Å) contacts with the 6-H and 6'-H hydrogens of bpy. Additional contact is observed between glutamine carboxyl oxygens and one the Cp* protons (2.46 Å). The fulvene complex $[Rh([Me_4Cp{=}CH_2)(bpy)OH_2]^+$ is 51 kJ/mol higher in energy than the hydroxide species, the specific interactions being still present (2.11 and 2.06 Å for the glycine oxygens and bpy hydrogens and 2.48 Å for the Cp* proton – glutamine oxygen). This complex is predicted to dissociate readily to Rh^I $[Rh([Me_4Cp{=}CH_2)(bpy)]^+$. For this system, the DFT calculation predicts the transfer of the –SH proton to glycine carboxylate and the specific interactions of the glutathione molecule with the rhodium complex are lost, apart from a single contact between a glutamine oxygen and bpy 5-H.

S.9. Synthesise and characterization of the complexes 1-12

Chelating ligands (2 - 2.5 mol equiv) were added to the appropriate chloride-bridged dimer $[(\eta^5\text{-}Cp^x)Rh/IrCl_2]_2$ in dry dichloromethane in a round-bottom flask. The reaction was stirred overnight at ambient temperature, and the solvent removed on a rotary evaporator to afford a crude powder which was dissolved in methanol and filtered. Excess NH_4PF_6 was then added and the solution placed in a freezer. The resulting product was collected by filtration and recrystallized from acetone or methanol. The pyridine complex $[(Cp^*)Rh(bpy)(py)](PF_6)_2$ (**12**) was synthesized by $AgNO_3$ treatment (1 mol equiv) of **1** in a 1:1 v/v mixture of $MeOH{:}H_2O$ followed by the reaction with excess pyridine (10 mol equiv) and NH_4PF_6 at ambient temperature.

$[(Cp^*)Rh(bpy)Cl]PF_6$ (1). $[(Cp^*)RhCl_2]_2$ (100.1 mg, 161.9 µmol), 2,2'-bpyridine (64.7 mg, 414.3 µmol). Recrystallization from acetone resulted in bright orange crystals. Yield: 126.6

mg (67.9 %). ^{1}H NMR (400 MHz, Acetone-d_6): δ_H 9.14 (d, 2H, J = 5.7 Hz), 8.67 (d, 2H, J = 8.1 Hz), 8.38 (t, 2H, J = 8.0 Hz), 7.95 (dd, 2H, J_1 = 8.0 Hz, J_2 = 5.7 Hz), 1.82 (s, 15H). Anal: Calc for $C_{20}H_{23}ClF_6N_2PRh$ C: 41.8, H: 4.03, N: 4.87; Found C: 41.85, H: 3.97, N: 4.84. ESI-MS: Calc for $C_{20}H_{23}ClN_2Rh$ $(M)^+$ 430.1 *m/z,* found 430.0 *m/z.*

[(Cp*)Ir(bpy)Cl]PF$_6$(2). [(Cp*)IrCl$_2$]$_2$ (51.0 mg, 64.1 µmol), 2,2'-bpyridyl (29.2 mg, 187.1µmol). Recrystallization from acetone resulted in bright orange crystals. Yield: 53.28 mg (59.9 %). ^{1}H NMR (400 MHz, MeOD-d_4): δ_H 9.13 (d, 2H, J = 6.2 Hz), 8.64 (d, 2H, J = 8.1 Hz), 8.42 (t, 2H, J = 8.0 Hz), 7.94 (dd, 2H, J = 6.0 Hz), 1.82 (s, 15H).

[(Cp^{xPh})Rh(bpy)Cl]PF$_6$ (3). [(Cp^{xPh})RhCl$_2$]$_2$ (101.3 mg, 136.5 µmol), 2,2'-bpyridine (56.0 mg, 358.5 µmol). Recrystallization from acetone resulted in bright orange crystals. Yield: 113.5 mg (65.2%). ^{1}H NMR (400 MHz, Acetone-d_6): δ_H 8.85 (d, 2H,J = 5.5 Hz), 8.70(d, 2H, J = 8.1 Hz), 8.37 (t, 2H, J = 8.4 Hz), 7.85 (t, 2H, J = 6.4 Hz), 7.76 (d, 2H, J = 6.6 Hz), 7.60 (m, 3H), 1.92 (s, 6H), 1.85 (s, 6H). Anal: Calc for $C_{25}H_{25}ClF_6N_2PRh$ + acetone C: 48.40, H: 4.50, N: 4.03; Found C: 49.89, H: 4.22, N: 3.93. ESI-MS: Calc for $C_{25}H_{25}ClN_2Rh$ $(M)^+$ 490.1 *m/z,* found 490.0 *m/z.*

[(Cp^{xPhPh})Rh(bpy)Cl]PF$_6$ (4). [(Cp^{xPhPh})RhCl$_2$]$_2$ (100.8 mg, 112.4 µmol) 2,2'-bpyridine (45.2 mg, 289.4 µmol). Recrystallization from acetone resulted in bright orange crystals. Yield: 87.2 mg (54.3 %). ^{1}H NMR (400 MHz, Acetone-d_6): δ_H 8.91 (d, 2H, J = 8.2 Hz), 8.89 (d, 2H, J = 8.4 Hz), 8.35 (t, 2H, J = 8.0 Hz), 7.87 (m, 6H), 7.79 (d, 2H, J = 7.6 Hz), 7.54 (t, 2H, J = 7.6 Hz), 7.45 (t, 1H, J = 7.6 Hz), 1.95 (s, 6H), 1.90 (s, 6H). Anal: Calc for $C_{31}H_{29}ClF_6N_2PRh$ C: 52.23, H: 4.10, N: 3.93; Found C: 52.73, H: 4.31, N: 3.70. ESI-MS: Calc for $C_{31}H_{29}ClN_2Rh$ $(M)^+$ 567.1 *m/z*, found 567.0 *m/z.*

[(Cp*)Rh(mbpy)Cl]PF$_6$ (5). [(Cp*)RhCl$_2$]$_2$ (49.6 mg, 80.2 µmol), 2,2'-bpyridyl (30 mg, 164.5 µmol). Recrystallization from acetone resulted in bright orange crystals. Yield: 68.1

mg (73.3 %). ^{1}H NMR (400 MHz, $CDCl_3$): δ_H 8.80 (s, 2H), 8.65 (d, 2H, J = 5.5 Hz), 7.56 (d, 2H, J = 5.8 Hz), 2.67 (s, 6H), 1.74 (s, 15H). Anal: Calc for $C_{22}H_{27}ClF_6N_2PRh$ C: 43.84, H: 4.51, N: 4.65; Found C: 42.62, H: 4.34, N: 4.70. ESI-MS: Calc for $C_{22}H_{27}ClN_2Rh$ $(M)^+$ 457.0 *m/z*, found 457.0 *m/z*.

$[(Cp^{xPh})Rh(mbpy)Cl]PF_6$ (6). $[(Cp^{xPh})RhCl_2]_2$ (51.7 mg, 69.7 µmol) 2,2'-bpyridyl (24.9 mg, 136.5 µmol). Recrystallization from acetone resulted in bright orange crystals. Yield: 68.1 mg (73.6%). ^{1}H NMR (400 MHz, $CDCl_3$): δ_H 8.88 (s, 2H), 8.35 (d, 2H, J = 6.6 Hz), 7.68 (d, 2H, J = 6.9 Hz), 7.56 (m, 3H), 2.65 (s, 6H), 1.85 (s, 6H), 1.76 (s, 6H). Anal: Calc for $C_{27}H_{29}ClF_6N_2PRh$ C: 48.78, H: 4.40, N: 4.21; Found. C: 47.29, H: 4.22, N: 3.99. ESI-MS: Calc for $C_{27}H_{29}ClN_2Rh$ $(M)^+$ 519.0 *m/z*, found 519.0 *m/z*.

$[(Cp^{xPhPh})Rh(mbpy)Cl]PF_6$ (7). $[(Cp^{xPhPh})RhCl_2]_2$ (49.6 mg, 55.3 µmol), 2,2'-bpyridyl (20.7 mg, 113.5 µmol). Recrystallization from acetone resulted in bright orange crystals. Yield: 53.4 mg (65.2 %) ^{1}H NMR (400 MHz, $CDCl_3$): δ_H 8.88 (s, 2H), 8.40 (d, 2H, J = 5.7 Hz), 7.76 (m, 4H), 7.67 (d, 2H, J = 7.7 Hz), 7.50 (t, 2H, J = 7.9 Hz), 7.45 (m, 1H), 7.41 (d, 2H, J = 6.5 Hz), 2.65 (s, 6H), 1.86 (s, 6H), 1.81 (s, 6H). Anal: Calc for $C_{33}H_{34}ClF_6N_2PRh$ C: 53.49, H: 4.49, N: 3. 78; Found C: 52.91, H: 4.35, N: 3.75. ESI-MS: Calc for $C_{33}H_{34}ClN_2Rh$ $(M)^+$ 595.0 *m/z*, found 595.0 *m/z*.

$[(Cp^*)Rh(phen)Cl]PF_6$ (8). $[(Cp^*)RhCl_2]_2$ (100.2 mg, 162.1 µmol), 1,10-phenanthroline (80.78 mg, 448.2 µmol). Recrystallization from acetone resulted in bright orange crystals. Yield: 138.7 mg (71.4 %). ^{1}H NMR (400 MHz, $CDCl_3$): δ_H 9.43 (d, 2H, J = 5.2 Hz), 8.71 (d, 2H, 8.1 Hz), 8.29 (dd, 2H, J_1 = 8.2 Hz, J_2 = 5.2 Hz), 8.1 (s, 2H), 1.89 (s, 15H). Anal: Calc for $C_{22}H_{23}ClF_6N_2PRh$ C: 44.13, H: 3.87, N: 4.68; Found C: 44.13, H: 3.79, N: 4.62. ESI-MS: Calc for $C_{22}H_{23}ClN_2Rh$ $(M)^+$ 453.1 *m/z* found 453.0 *m/z*.

[(Cp^{xPh})Rh(phen)Cl]PF$_6$ (9).[(Cp^{xPh})RhCl$_2$]$_2$ (124.0 mg, 167.1 µmol), 1,10-phenanthroline (83.6 mg, 463.9 µmol). Recrystallization from acetone resulted in bright red crystals. Yield: 130.3 mg (59.0 %). ^{1}H NMR (400 MHz, CDCl$_3$): δ$_H$ 9.08 (d, 2H, J = 5.2 Hz), 8.69 (d, 2H, J = 8.2 Hz), 8.12 (m, 4H), 7.78 (d, 2H, J = 7.6 Hz), 7.61 (m, 3H), 2.06 (s, 6H), 1.85 (s, 6H). Anal: Calc for $C_{27}H_{25}ClF_6N_2PRh$ + MeOH C: 48.54, H: 4.22, N: 4.04; Found C: 47.68, H: 4.09, N: 4.17. ESI-MS: Calc for $C_{27}H_{25}ClN_2Rh$ (M)$^+$ 515.1 *m/z*, found 515.0 *m/z*.

[(Cp^{xPhPh})Rh(phen)Cl]PF$_6$ (10). [(Cp^{xPhPh})RhCl$_2$]$_2$ (100.7 mg, 112.3 µmol), 1,10-phenanthroline (55.8 mg, 309.6 µmol). Yield: 109.8 mg (66.3 %) ^{1}H NMR (400 MHz, CDCl$_3$): δ$_H$ 9.11 (d, 2H, J = 5.2 Hz), 8.69 (d, 2H, J = 8.1 Hz), 8.13 (dd, 2H, J$_1$ = 8.1 Hz, J$_2$ = 5.2 Hz), 8.12 (s, 2H), 7.86 (d, 2H, J = 8.2 Hz), 7.82 (d, 2H, J = 8.2 Hz), 7.70 (d, 2H J = 7.4 Hz), 7.53 (t, 2H, J = 7.4), 7.45 (t, 1H, J = 7.4 Hz), 2.06 (s, 6H), 1.88 (s, 6H). Anal: Calc for $C_{33}H_{29}ClF_6N_2PRh$ + MeOH C: 53.11, H: 4.33, N: 3.64; Found C: 54.37, H: 4.34, N: 3.49. ESI-MS: Calc for $C_{33}H_{29}ClN_2Rh$ (M)$^+$ 591.1 *m/z*, found 591.1 *m/z*.

[(Cp*)Rh(en)Cl]PF$_6$ (11). [(Cp*)RhCl$_2$]$_2$ (51.3 mg, 83.0 µmol), ethylenendiamine (9.88 mg, 0.16 mmol, 11 µl). Bright yellow crystals were collected. Yield: 42.3 mg (78.3 %). ^{1}H NMR (400 MHz, CDCl$_3$): δ$_H$ 5.91 (s, 2H), 3.05 (s, 2H), 2.88 (s, 2H), 2.74 (s, 2H), 1.90 (s, 15H). Anal: Calc for $C_{12}H_{23}ClF_6N_2PRh$ C: 27.91, H: 4.61, N: 6.21; Found C: 28.11, H: 4.81, N: 5.95. ESI-MS: Calc for $C_{12}H_{23}ClN_2O_2Rh$ (M)$^+$ 333.0 *m/z*, found 333.0 *m/z*.

[(Cp*)Rh(bpy)(py)](PF$_6$)$_2$ (12). Yield: 47.9 %. ^{1}H NMR (400 MHz, Acetone-d$_6$): δ$_H$ 9.46 (d, 2H, J = 4.8 Hz), 8.57 (m, 4H), 8.37 (t, 2H, J = 8.0 Hz), 8.03 (t, 2H, J = 8.0 Hz), 8.95 (t, 1H, J = 8.0 Hz), 7.48 (t, 2H, J = 8.0 Hz), 1.70 (s, 15H). ESI-MS: Calc for $C_{25}H_{28}N_3Rh$ (M)$^{2+}$ 236.5664 *m/z* found 236.5664 *m/z*. Crystals of **12·0.5H$_2$O** were obtained from an acetone solution of complex **12** on slow evaporation of the solvent at ambient temperature.

S10. Aqueous solution studies

S.10.1 Hydrolysis and pK_a determinations

Solutions of complexes **1-11** (1.4 mM, in 20 % MeOD-d_4/ 80 % D_2O v/v) were prepared and ^{1}H NMR spectra at 310 K was recorded at time 0 (<10 min) and 24 h. The samples were incubated at 310K.

An aqueous solution of the chloride complex (**1-11**, 1.4 mM, in 20 % MeOD-d_4/ 80 % D_2O v/v) was treated with silver nitrate (0.95 mol. equiv.) and stirred overnight at room temperature. ^{1}H NMR spectra were recorded after filtration of the samples through Celite to remove the silver chloride formed. Complexes **1**, **3-11** showed fast hydrolysis at 310 K, reaching equilibrium in <10 min. Complete conversion of chloride complex **11** to the aqua adduct was observed, whereas **1**, and **3-10** reached equilibrium with 30-60% formation of the aqua species.

Solutions of the complexes **1**, **8** and **11** (1.4 mM, D_2O) were prepared and treated with 0.95 mol equiv silver nitrate. The reaction mixture was then filtered through Celite to obtain a solution of the corresponding aqua adducts. Changes in the chemical shifts of ligand protons of the aqua adducts for complexes **1**, **8** and **11** with the pH* (pH meter reading) over a range from 2 to 12 were followed by ^{1}H NMR spectroscopy. Solutions of KOH or $HClO_4$ in D_2O were used to adjust the pH*. ^{1}H NMR spectra were recorded at 298 K on a Bruker AV III 600 spectrometer. The data were fitted to the Henderson–Hasselbalch equation using Origin 7.5.

S.10.2 Cyclopentadienyl deuteration by ^{1} H NMR spectroscopy

Solutions of complexes **1-11** in MeOD-d_4/D_2O (3/5 v/v) were prepared (1.4 mM). The solutions were then treated with 0.95 mol equiv $AgNO_3$ in order to obtain the corresponding aqua species. After 12-24 h reaction, the complexes were filtered through Celite and ^{1}H NMR

spectra were recorded. Samples were incubated at ambient temperature or 310 K. Samples from **1-10** were also analyzed by FTICR MS after 72 h.

A solution of complex **1** (1.4 mM) in MeOH/ H_2O (3/5 v/v) was also prepared and treated with 0.95 mol equivAgNO_3. A ^{1}H NMR spectrum recorded after 24 h incubation at ambient temperature showed a decrease in intensity, splitting, and broadening of the Cp^x peaks, and FT-ICR MS spectra recorded every 10 min for 80 min and after 27 and 42 h, showed gradual deuteration of Cp^x ring methyls.

S.10.3 Kinetic experiments for the deuteration of cyclopentadienyl ligands

A solution of 1.4 mM of complex **1** in MeOD-d_4/D_2O (5/3, v/v) was treated with 0.95 mol equiv AgNO_3. After 50 min incubation, the AgCl precipitate was filtered through Celite and the sample was placed in a 5 mm NMR tube. ^{1}H NMR spectra were then recorded every 410 sec.

A second experiment using 1.4 mM solutions of complex **1** in MeOD-d_4/D_2O (3/5, v/v) was performed. The sample was treated with 0.95 mol equiv AgNO_3 and left to react for 20 min before filtration of the AgCl precipitate. The samples were analyzed by FT-ICR MS. Samples were taken every 10 min for a period of 80 min, when the reaction seemed to have reached equilibrium; subsequent sampling occurred hourly and then after 27 and 42 h.

S.10.4 Effect of complex hydrolysis on the deuteration of cyclopentadienyl ligands

Two solutions of complex **1** were prepared in 60% MeOD-d_4/ 40% D_2O, v/v. The first solution was left to hydrolyze while the second solution was reacted with silver nitrate (0.95 mol equiv) to promote formation of the aqua complex. After 12 h incubation at ambient temperature, the samples were filtered and the solvent removed on a rotary evaporator to obtain yellow powders. As a control, a third sample of complex **1** (1.4 mM) was prepared in

MeOH/H_2O (3.5/1, v/v), and reacted with 0.95 mol equiv $AgNO_3$. The three samples were then re-dissolved in d_6-acetone. ^1H NMR spectra of the samples were recorded.

A series of experiments using complex **1** and NaCl in MeOD-d_4 were also performed. A sample of complex **1** was saturated with NaCl, while a second sample was prepared in the absence of added chloride. ^1H NMR spectra of the samples were recorded after ca. 16 h of incubation at ambient temperature.

S.10.5 Effect of methanol on the deuteration of cyclopentadienyl ligands

A series of experiments using different amounts of methanol-d_4 was performed. In these experiments, complex **1** was dissolved in MeOD-d_4/D_2O (0, 20, 60 and 100% MeOD-d_4). The solutions were not treated with silver nitrate. The reaction mixture was incubated at ambient temperature and ^1H NMR spectra were recorded at various intervals over a period of 20 h. Methanol increased the extent of deuteration of **1** (Figure S6), increasing from 6% in D_2O, to 20% in 40% D_2O/60% MeOD-d_4 to 66% in MeOD-d_4 alone, after ca. 19 h at 293 K.

S.10.6 Effect of pH on the deuteration of cyclopentadienyl ligands

Two solutions of complex **1** (1.4 mM, 60% MeOD-d_4/ 40% D_2O v/v) were prepared. The first sample was then treated with 50 µL of NaOD solution (40 wt% in D_2O, 99+ atom% D) and placed in a 5 mm NMR tube. A ^1H NMR spectrum of the sample was recorded at ambient temperature.

The second solution of complex **1** in 60% MeOD-d_4/40% D_2O v/v was then treated with 50 µL of DNO_3 solution (65 wt% in D_2O, 99 atom% D). ^1H NMR spectra were recorded after addition of the acid and after 1, 3 and 12 h incubation at ambient temperature.

Complex **1** was also dissolved in D_2O (0.8 mM) and treated with 10 µL of NaOD solution (40 wt% in D_2O, 99+ atom% D). The reaction was followed by ^1H NMR at ambient temperature.

S.10.7 Reversibility of the H/D exchange reactions

A solution of 1.4 mM of complex **1** in D_2O was prepared and treated with 0.95 mol equiv $AgNO_3$. After 20 min incubation, the solution was filtered and the samples were analysed by FT-ICR MS at t_0 and then hourly up to 14 h; subsequent samples were taken after 24 h and 7 d. The solution was then freeze-dried to give solid D_{15}**1**.

A solution of 1.4 mM of complex D_{15}**1** in H_2O was prepared and analysed by FT-ICR MS at t_0 and then hourly up to 14 h, and after 24 h and 7 d. FT-ICR mass spectra showed only a small amount of re-protonation of D_{15}**1** after 7 days' incubation. This suggests that the deuterated compounds are relatively stable towards back exchange in water.

S11. Reaction of complex 1 with N-methylmaleimide

[(η^5-Cp*)Rh(bpy)Cl]PF_6 (**1**, 22 mg, 0.04 mmol) was placed in a round-bottom flask and 60% methanol-d_4/40% D_2O (1 mL) was added. After addition of the N-methylmaleimide (2–4 mol equiv), the reaction was stirred for various times at 310 K. ^1H NMR spectra and the ESI-MS were recorded to monitor the reaction.

S12. Reaction of complex 1 with isoprene: formation of complex 13

Isoprene (10 mol equiv) was added to [(η^5-Cp*)Rh(bpy)Cl]PF_6 (**1**, 22 mg, 0.04 mmol) in 60% methanol-d_4/40% D_2O v/v, 1 mL) in a round-bottom flask. The reaction was stirred for various times at 310 K and ESI-MS and HRMS of the reaction mixture were recorded to monitor the reaction. The yield of the complex **13** was ca. 21% in the reaction mixture. The product, the Rh^I complex, **13**, was fully characterized by LCMS, HRMS, MS/MS and *in situ*

1D and 2D NMR. The same reaction was also repeated in 1% DMSO-d_6/9% D_2O/ 90% RPMI-1640 cell culture medium supplemented with 10% of fetal calf serum.

S13. Reaction of complex 1 with conjugated (9Z,11E)-linoleic acid: formation of complex 14

Conjugated (9Z,11E)-linoleic acid (10 mol equiv) was added to [(η^5-Cp*)Rh(bpy)Cl]PF_6 (**1,** 22 mg, 0.04 mmol) in 60% methanol-d_4/40% D_2O v/v (1 mL) in a round-bottom flask. The reaction was stirred for various times at 310 K. ESI-MS and HRMS of the reaction mixture were recorded to monitor the reaction. The product, the Rh^I complex **14**, was characterized by HRMS and MS-MS. The yield of the complex **14** was ca. 23% in the reaction mixture. The reversibility of this cycloaddition reaction was apparent from its dissociation on dilution as indicated by ESI-MS data (Figure. S24). The reaction was repeated in 1% DMSO-d_6/ 9% D_2O/ 90% RPMI-1640 cell culture medium supplemented with 10% of fetal calf serum.

S14. Reaction of complex 10 with isoprene: formation of complex 15

Isoprene (10 mol equiv) was added to [(Cp^{xPhPh})Rh(phen)Cl]PF_6 (**10**, 15 mg, 0.02 mmol) in 60% methanol-d_4/40% D_2O v/v, 1 mL) in a round-bottom flask. The reaction was stirred for various times at 310 K and ESI-MS and HRMS of the reaction mixture were recorded to monitor the reaction. The yield of the complex **15** was ca. 27% in the reaction mixture. The product, the Rh^I complex, **15**, was fully characterized by LCMS, HRMS, MS/MS.

S15. ^{2}H NMR of [($D_{15}\eta^5$-Cp*)Rh(bpy)Cl]PF_6

[(D_{15}-η^5-Cp*)Rh(bpy)Cl]PF_6 was obtained by full deuteration of [(η^5Cp*)Rh (bpy)Cl]PF_6 in 60% MeOD-d_4/40% D_2O over 72 h at 310 K. 76.77 MHz ^{2}H NMR spectra were recorded in 60% methanol/40% H_2O with 2 µL of acetone-d_6 in 500 µL solution as internal standard or in

60% methanol/40% RPMI-1640 cell culture medium supplemented with 10% of fetal calf serum.

Supplementary Tables

Table S1. Crystallographic data for complexes **1, 3, 4, 6, 7, 9**, **10** and **12**.

	**1**	**3·2Me$_2$CO**	**4·Me$_2$CO**
Formula	$C_{20}H_{23}ClF_6N_2PRh$	$C_{31}H_{37}ClF_6N_2O_2PRh$	$C_{34}H_{35}ClF_6N_2OPRh$
FW	574.73	752.96	770.97
Temp (K)	296 (2)	120 (2)	150 (2)
Crystal system	orthorhombic	triclinic	triclinic
Space group	Pmn2(1)	P-1	P-1
a(A)	12.9507 (9)	8.51582 (16)	8.40215(19)
b(A)	8.3100 (7)	13.5384 (4)	12.9603(3)
c(A)	10.5094 (8)	14.2569 (3)	15.3920(4)
α(°)	90	94.5969 (19)	80.000(2)
β(°)	90	92.9146 (16)	86.442(2)
γ(°)	90	92.6758 (18)	86.175(2)
U(A^3)	1131.03 (15)	1634.16 (6)	1644.78(7)
λ (A)	0.71073	0.71073	1.54184
Z	2	2	2
D_{calc}(mg/cm^3)	1.688	1.53	1.557
μ(mm^{-1})	1.003	0.719	5.975
F(000)	576	768	784
Reflections measured	5948	20346	18378
Indep reflections	2381	9604	6984
R1 [I>2σ(I)][a]	0.0303	0.0382	0.0332
wR_2(all data)[b]	0.0758	0.832	0.0897
CCDC no.	1420047	1420049	1420052

	**6**	**7·Me_2CO**
Formula	$C_{27}H_{29}ClF_6N_2PRh$	$C_{36}H_{39}ClF_6N_2PRh$
FW	664.85	799.02
Temp (K)	296 (2)	150 (2)
Crystal system	monoclinic	monoclinic
Space group	P2(1)/n	P2(1)/n
a(Å)	15.9555 (8)	8.0233 (4)
b(Å)	11.5878 (4)	28.1842 (13)
c(Å)	17.1341 (8)	16.1096 (8)
α(°)	90	90
β(°)	116.061 (6)	94.393 (4)
γ(°)	90	90
U(A^3)	2846.6 (2)	3632.2 (3)
λ (Å)	0.71073	0.71073
Z	4	4
D_{calc}(mg/cm^3)	1.551	1.469
μ(mm^{-1})	0.809	0.649
F(000)	1344	1632
Reflections measured	29284	22985
Indep reflection	8079	9993
R1 [I>2σ(I)][a]	0.378	0.0706
wR_2(all data)[b]	0.0999	0.1915
CCDC no.	1420046	1420050

	9·MeOH	10·Me_2CO	12·0.5H_2O
Formula	$C_{28}H_{29}ClF_6N_2PRh$	$C_{36}H_{35}ClF_6N_2OPRh$	$C_{25}H_{28}F_{12}N_3O_{0.5}P_2Rh$
FW	692.86	794.99	771.35
Temp (K)	150 (2)	568 (2)	150(2)
Crystal system	triclinic	triclinic	triclinic
Space group	P-1	P-1	P-1
a(Å)	8.20707 (8)	8.7508 (4)	9.84864(19)
b(Å)	10.08952 (12)	14.4955 (6)	10.1712(3)
c(Å)	17.2032 (3)	14.5101 (6)	16.1627(3)
α(°)	82.3397 (11)	84.910 (3)	101.363(2)
β(°)	81.4575 (11)	74.415 (4)	90.9323(17)
γ(°)	84.0175 (9)	82.382 (3)	109.801(2)
U(A^3)	1390.93	1754.53	1487.41(6)
λ (Å)	0.71073	0.71073	1.54184
Z	2	2	2
D_{calc}(mg/cm^3)	1.654	1.505	1.722
μ(mm^{-1})	0.834	0.672	6.585
F(000)	700	808	772.0
Reflections measured	86143	18176	44172
Indep reflection	9499	9416	6273
R1 [I>2σ(I)][a]	0.0281	0.0415	0.0482
wR_2(all data)[b]	0.0681	0.1162	0.1266
CCDC no.	1420051	1420048	1569283

Table S2. Selected bond lengths (Å) angles (º) for complexes **1, 3, 4, 6, 7, 9**, **10** and**12**.

	**1**	**3**	**4**
Rh1-N' (Å)	2.105 (3)	2.098 (19)	2.109 (2)
Rh1-N (Å)	2.105 (3)	2.1076 (17)	2.099 (2)
Rh1-Cl1 (Å)	2.3972 (14)	2.4027 (5)	2.3859 (6)
N'-Rh1-N (º)	76.68 (16)	76.70 (7)	76.63 (8)
N'-Rh1-Cl1 (º)	86.74 (9)	88.82 (5)	87.46 (6)
Cl1-Rh1-N (º)	86.74 (9)	88.64 (5)	88.18 (6)
	**6**	**7**	**9**
Rh1-N' (Å)	2.1064 (13)	2.116 (2)	2.127 (2)
Rh1-N (Å)	2.1156 (12)	2.131 (2)	2.102 (2)
Rh1-Cl1 (Å)	2.4011 (4)	2.3919 (7)	2.3773 (7)
N'-Rh1-N (º)	77.96 (5)	77.48 (8)	76.40 (8)
N'-Rh1-Cl1 (º)	86.77 (4)	88.93 (7)	88.00 (6)
Cl1-Rh1-N (º)	85.75 (4)	85.54 (7)	86.40 (6)
	**10**		**12**
Rh1-N' (Å)	2.107 (4)	Ru1-N' (Å)	2.106(4)
Rh1-N (Å)	2.101 (4)	Ru1-N (Å)	2.105(3)
Rh1-Cl1 (Å)	2.3855 (13)	Ru1-N(py) (Å)	2.127(4)
N'-Rh1-N (º)	77.12 (15)	N'-Rh1-N (º)	77.05(16)
N'-Rh1-Cl1 (º)	85.03 (11)	N'-Rh1-N(py) (º)	85.45(16)
Cl1-Rh1-N (º)	85.43 (11)	N(py)-Rh1-N (º)	86.67(16)

Table S3. IC_{50} values for complexes **1, 2** and **4-11** in A2780 human ovarian cancer cells, extent of hydrolysis (equilibria reached in <10 min), and pK_a* values for aqua adducts.

Complex	IC_{50} (μM)	Hydrolysis[a] (%)	pK_a* [b]
**1**	64 ± 1	56	8.84 ± 0.08
**2**	>100	-	6.94
**3**	-	38	-
**4**	36.07 ± 0.07	49	-
**5**	65.1 ± 0.9	68	-
**6**	47.3 ±0.8	42	-
**7**	5.63 ± 0.23	32	-
**8**	17.8 ± 0.6	59	8.86 ± 0.06
**9**	57 ± 1	39	-
**10**	14.68 ± 0.05	48	-
**11**	>100	100	9.35 ± 0.06

[a] Hydrolysis in 20% MeOD/ 80% D_2O (v/v) at 310 K;
[b] pK_a in D_2O at 298 K;

Table S4. Comparison of the optimized structures of Rh and Ir complexes as $[(Cp^{*})M(bpy)OH]$ and $[M([Me_4Cp{=}CH_2]^{2-})(bpy)H_2O]$ isomers. All values given in Å. It is noteworthy that the TPPSh/QZVP method yields structures which reveal the shift of [M(Cp)(bpy)OH] to $M([Me_4Cp{=}CH_2]^{2-})(bpy)H_2O$ transition, indicating stronger elongation of the M-O bond and "upward shift" of the dianion regarding the bpy plane, similar to that observed for the ethylenediamine complex.

M(Cp*)(bpy)OH

	Rh		Ir	
	**CAM-B3LYP**[a]	**TPSSh**[b]	**CAM-B3LYP**	**TPSSh**
M - N(bpy)[c]	2.106	2.118	2.117	2.112
M - O	2.017	2.013	2.039	2.033
$H(CH_3)$ - O	2.165	2.212	2.227	2.278

$M([Me_4Cp{=}CH_2]^{2-})(bpy)H_2O$

	Rh		Ir	
	**CAM-B3LYP**	**TPSSh**	**CAM-B3LYP**	**TPSSh**
M - N(bpy)[c]	2.112	2.097	2.096	2.089
M - O	2.303	2.458	2.289	2.479
$H(H_2O)$ - $C(=CH_2)$	2.117	2.181	2.091	2.120

[a]CEP-31G basis set; [b]QZVP basis set; [c]averaged value.

Table S5. Calculated (CAM-B3LYP/CEP-31G) electronic energy differences (kJ/mol) between Rh and Ir complexes as [M(Cp*)(L)OH] and [M([$Me_4Cp=CH_2$]$^{2-}$)(L)H_2O] isomers for different L ligands. Note the nearly constant offset of 35-40 kJ/mol for the Ir series, compared to the Rh series. The trend in the calculated energy difference implies increased stabilization of the [M([$Me_4Cp=CH_2$]$^{2-}$)(L)H_2O] isomers with increasing π-acceptor properties of the chelating ligand L. It is noteworthy that the overall number of 15 methyl group protons that may be transferred to oxygen atoms brings about an entropy contribution of Rln15 = 22.5 e.u. to the free enthalpy of formation of [M([$Me_4Cp=CH_2$]$^{2-}$)(L)H_2O] isomers, amounting to ca 7 kJ/mol at 296 K.

**L**[a]	**Rh**	**Ir**
en	60	96
(pyr)$_2$	57	97
pic	51	92
2,9-Me-phen	51	91
3,3‘ Me-bpy	36	72
3-Me- bpy	37	72
bpy	35	70
phen	33	69
pyr imine	30	67
bpyrimidine	29	66
diimine	22	58
diphenim	21	56
4,5-CNdiphenim	13	-

[a] en – 1,2-ethylenediamine; py–pyridine; pic – 2-aminomethyl pyridine; 2,9-Me-phen – 2,9 dimethyl-1,10-phenantroline; 3,3’.Me-bpy – 3,3’.dimethyl-2,2’-bpyridine; 3-Me- bpy -3-methyl-2,2’-bpyridine; bpy – 2,2’-bpyridine; phen – 1,10-phenantroline; pyr imine – 2-methylimino-pyridine; bpyrimidine – 2,2′-bpyrimidine, diimine – 1,2-ethylenediimine, diphenim – 1,2-phenylenediimine, 4,5-CNdiphenim – 4,5-dicyano-1,2-phenylenediimine.

Table S6. HRMS and MS-MS data for [4+2] cyclo-addition adduct of complex **1** with isoprene formed via a fulvene intermediate.

Chemical structure	Molecular formula	Theoretical Mass	Observed Mass from HRMS
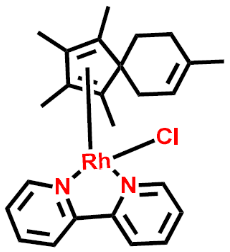	$C_{25}H_{31}ClN_2Rh$	497.1255 $(M+H)^+$	497.1225
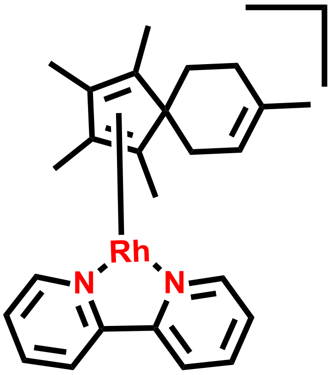	$C_{25}H_{30}N_2Rh$	461.1459 $(M)^+$	461.1459
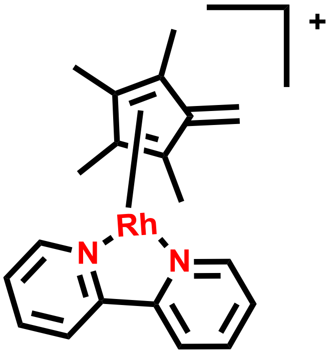	$C_{20}H_{22}N_2Rh$	393.0826 $(M)^+$	398.0826
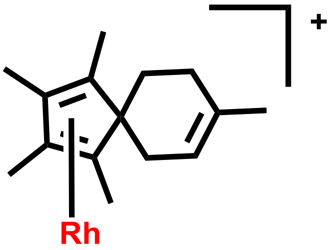	$C_{15}H_{22}Rh$	305.0764 $(M)^+$	305.0764

Table S7. HRMS and MS-MS data for [4+2] cyclo-addition adduct of complex **1** with conjugated (9Z,11E)-linoleic acid formed via a fulvene intermediate.

Chemical structure	Molecular formula	Theoretical Mass	Observed Mass from HRMS
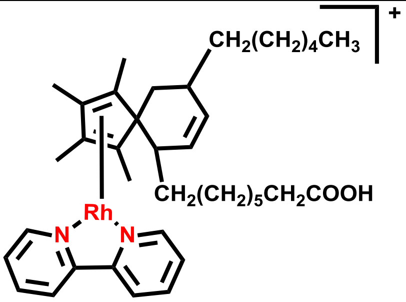	$C_{38}H_{54}N_2O_2Rh$<br><br>$C_{38}H_{53}DN_2O_2Rh$ (one deuterium incorporation, D_1**14**)	673.3235 $(M)^+$<br><br>674.3298(D_1**14**)$^+$	673.3235<br><br>674.3298
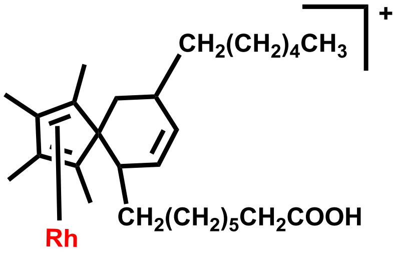	$C_{28}H_{46}O_2Rh$	517.2570 $(M)^+$	517.2570
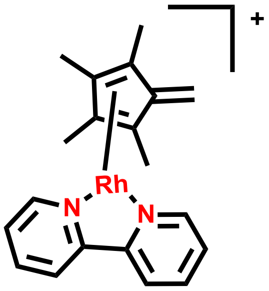	$C_{20}H_{22}N_2Rh$	393.0826 $(M)^+$	398.0826

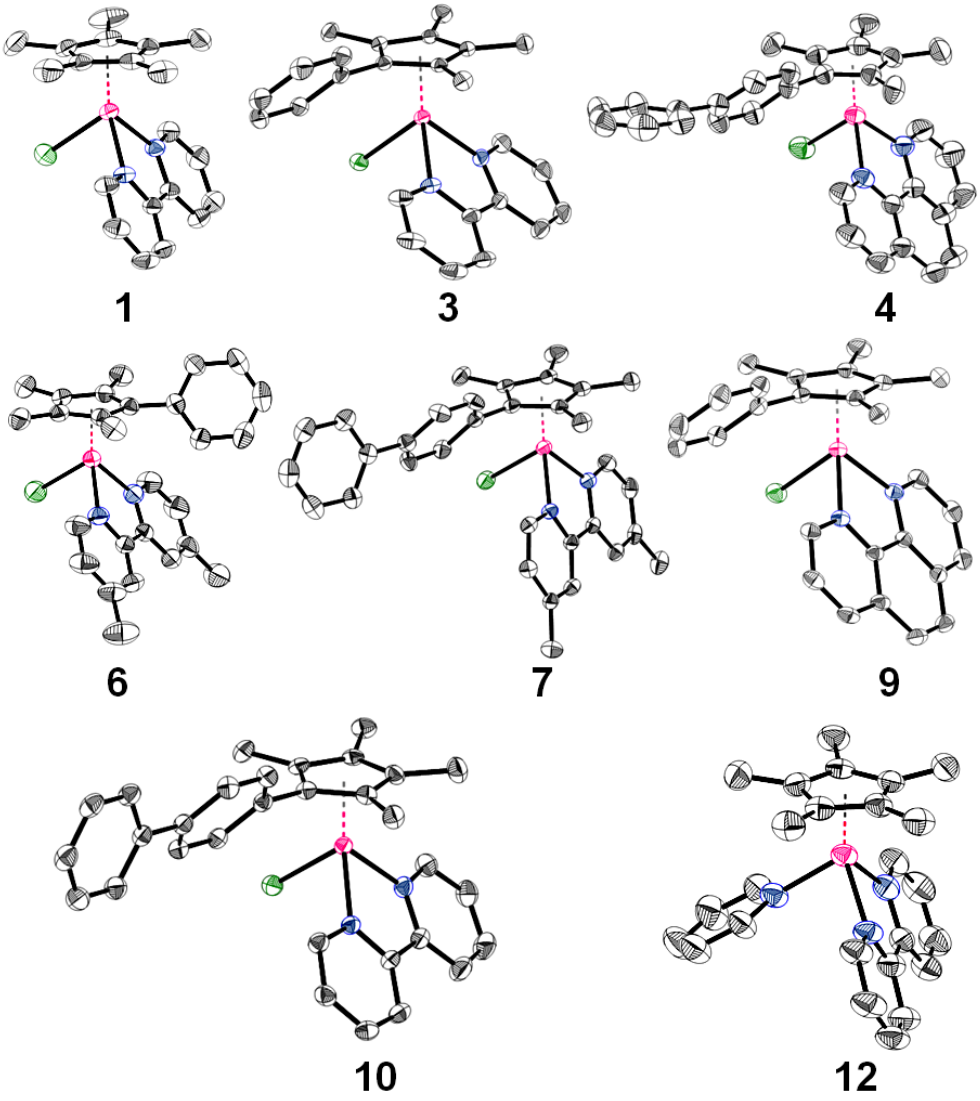

Figure S1 labels: **1**, **3**, **4**, **6**, **7**, **9**, **10**, **12**

Figure S1. X-ray crystal structures of complexes **1**, **3**, **4**, **6**, **7**, **9**, **10** and **12**. All C-H hydrogen atoms, counter-ions and solvent molecules have been omitted for clarity.

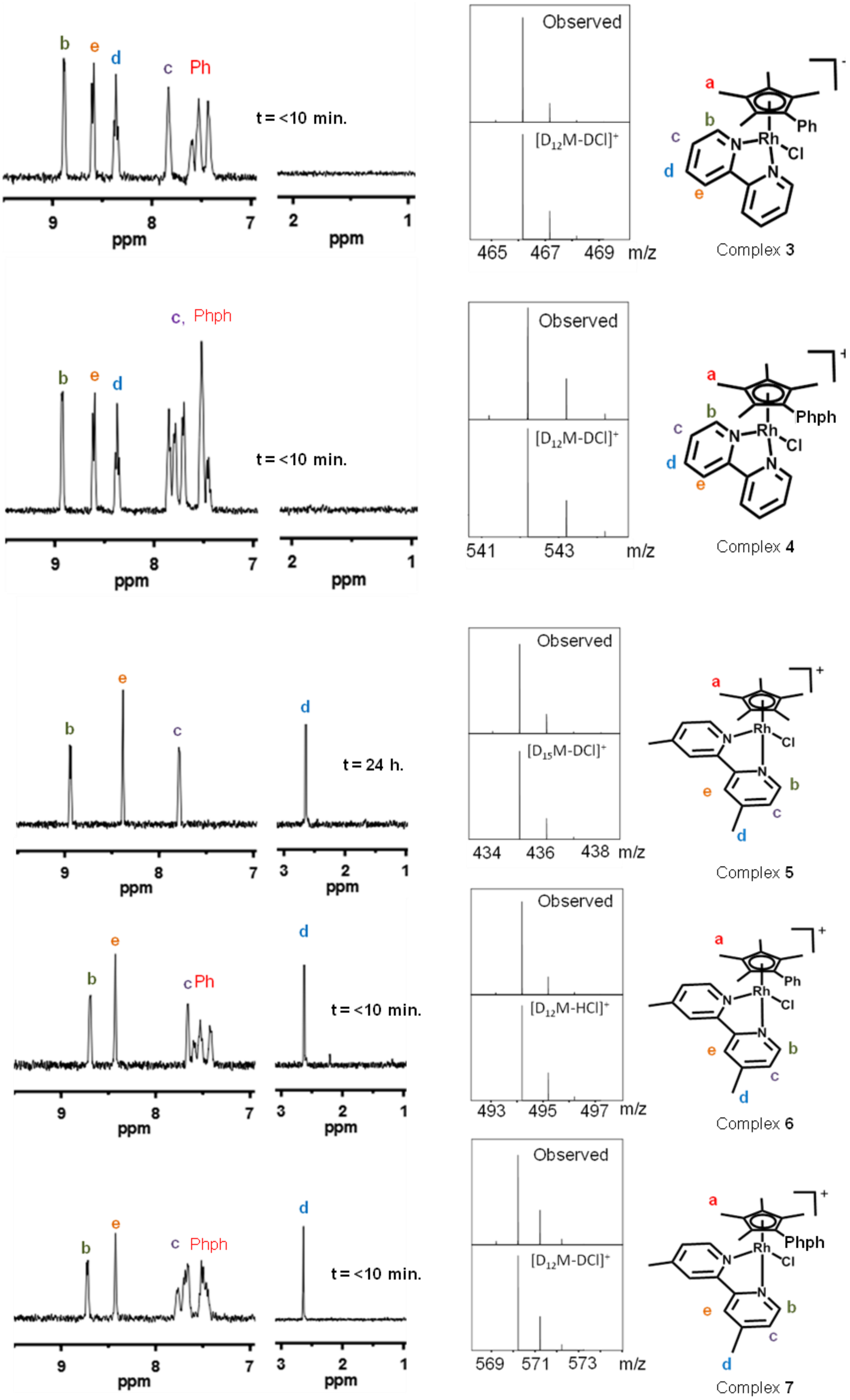
b e d c Ph
t = <10 min.
9 8 7 ppm
2 1 ppm
Observed
[D12M-DCl]+
465 467 469 m/z
a Ph Rh Cl N b c d e
Complex 3
c, Phph
b e d
t = <10 min.
9 8 7 ppm
2 1 ppm
Observed
[D12M-DCl]+
541 543 m/z
a Phph Rh Cl N b c d e
Complex 4
e b c d
t = 24 h.
9 8 7 ppm
3 2 1 ppm
Observed
[D15M-DCl]+
434 436 438 m/z
a Rh Cl N e b c d
Complex 5
e b c Ph d
t = <10 min.
9 8 7 ppm
3 2 1 ppm
Observed
[D12M-HCl]+
493 495 497 m/z
a Ph Rh Cl N e b c d
Complex 6
e b c Phph d
t = <10 min.
9 8 7 ppm
3 2 1 ppm
Observed
[D12M-DCl]+
569 571 573 m/z
a Phph Rh Cl N b e c d
Complex 7

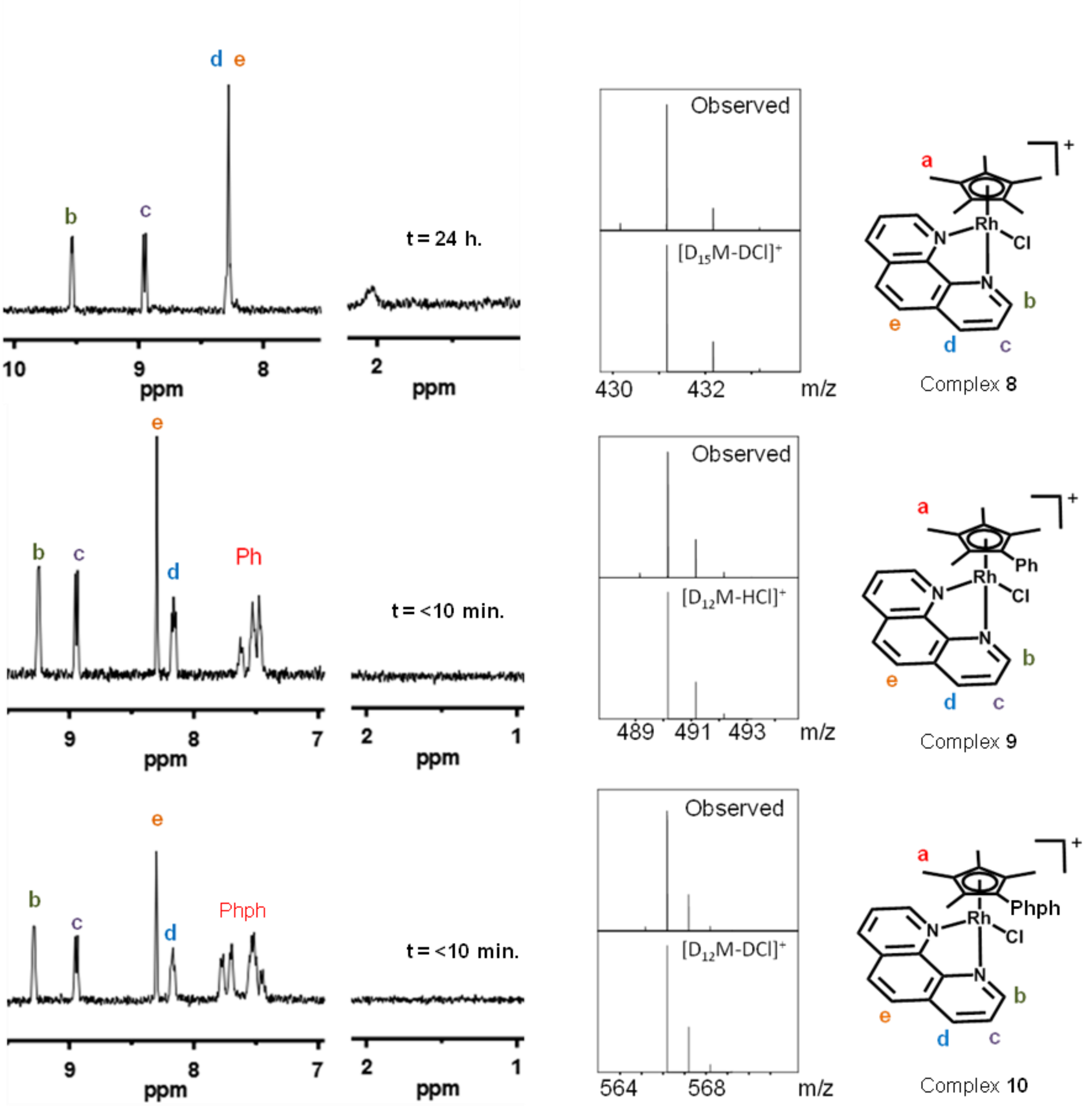

Figure S2. Effect of the chelated ligand on deuteration of methyls in Cp*, Cp^{xPh} and $Cp^{xPhPh}Rh^{III}$ complexes. ^{1}H NMR corresponding to complexes **3-7** (upper panel) and **8-10** (lower panel) after reaction with silver nitrate in 60% MeOD-d_4/40% D_2O for 24 h. FT-ICR mass spectra recorded after 72 h incubation. Deuteration of the Cp* in the rhodium complexes is observed.

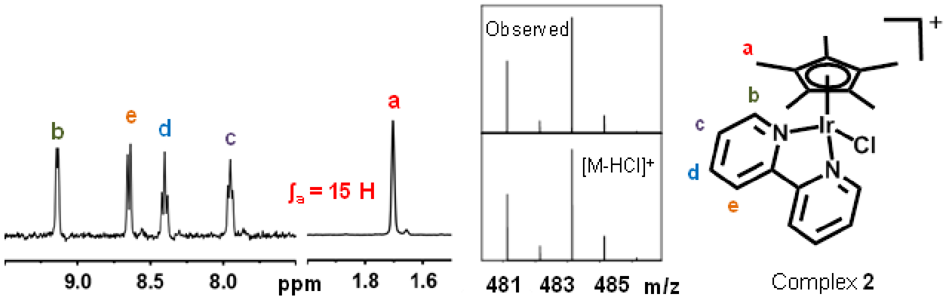

Figure S3. ^{1}H NMR spectra of complex **2** after reaction with $AgNO_3$ in 60% MeOD-d_4/40% D_2O for 24 h. The corresponding FT-ICR mass spectrum was recorded after 72 h incubation. No deuteration of the Cp* methyls is observed for this Ir^{III} complex.

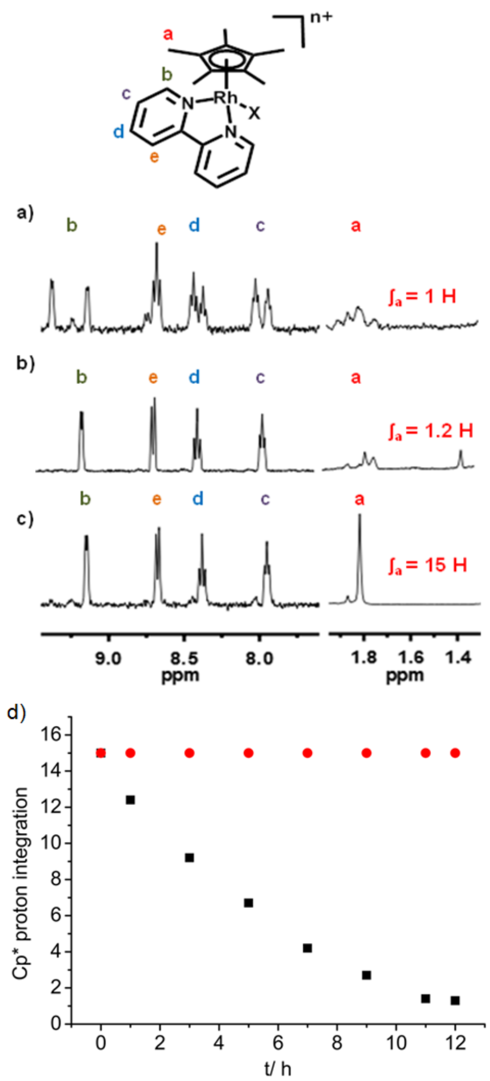

Figure S4. Effect of Ag^I ions on deuteration of Cp* in complex **1**. ^1H NMR spectra in acetone-d_6 of the product from incubation of complex **1** for 12 h at ambient temperature in methanol/water and in the absence or presence of Ag^I. X = H_2O or Cl, n = 1 or 2. a) Complex **1** incubated MeOD-d_4 / 40% D_2O (deuteration). Two sets of peaks corresponding to the chlorido and the aqua complexes can be observed. b) Complex **1** treated with $AgNO_3$ in 60% MeOD-d_4/40% D_2O (deuteration). c) Complex **1** treated with $AgNO_3$ in 60% CH_3OH/40% H_2O (no deuteration). d) Time dependent change in Cp* proton integration after treatment of complex **1** with $AgNO_3$ in 60% MeOD-d_4/40% D_2O (■) and in 60% CH_3OH/40% H_2O (●). The pattern of residual methyl ^1H peaks is influenced by small isotope shifts and by ^1H-D couplings in partially deuterated methyls.

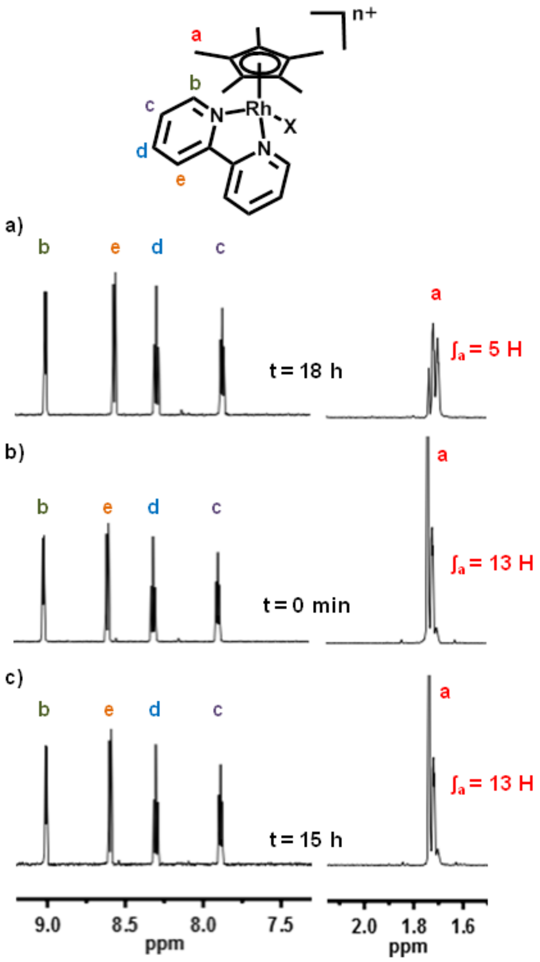

Figure S5. Effect of Cl^- ions on the deuteration of Cp* in complex **1**. ^{1}H NMR spectra of complex **1** incubated at ambient temperature in MeOD-d_4 in the presence or absence of excess chloride. X = H_2O or Cl, n = 1 or 2. a) After incubation for 18 h in the absence of NaCl. Integration of peak a corresponds to 5H. b) Immediately before addition of NaCl. Integration of a corresponds to 13H. c) After incubation for 15 h after addition of NaCl. Integration of a corresponds to 13H.

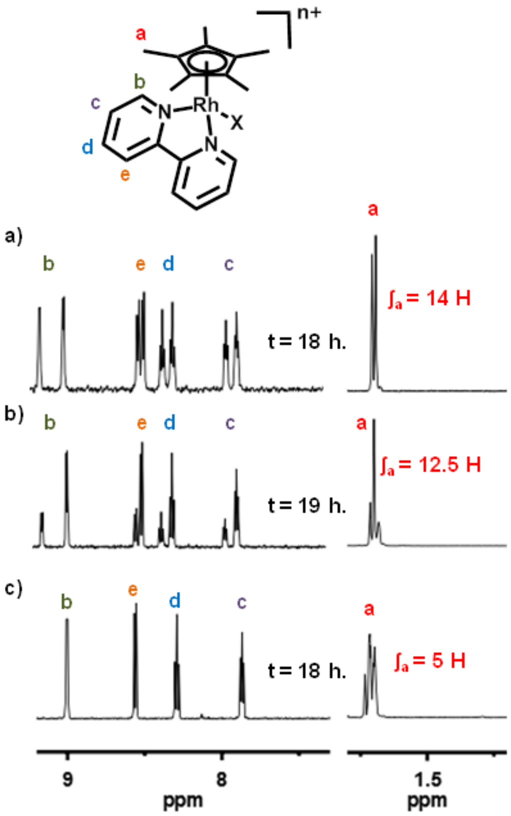

Figure S6. Effect of methanol concentration on the deuteration of Cp* in complex **1**. ^{1}H NMR spectra of complex **1** incubated at ambient temperature in MeOD-d$_4$ / D_2O. X = H_2O or Cl, n = 1 or 2. a) Complex in 20% MeOD-d$_4$/80 % D_2O. Two sets of peaks are observed corresponding to the chloride and aqua species. Integration of "a" corresponds to 14H. b) Complex in 60% MeOD-d$_4$/40% D_2O. Two sets of peaks are observed corresponding to the chloride and aqua species. Integration of "a" correspond to 12.5H. c) Complex in 100% MeOD-d$_4$. Integration of "a" corresponds to 5H.

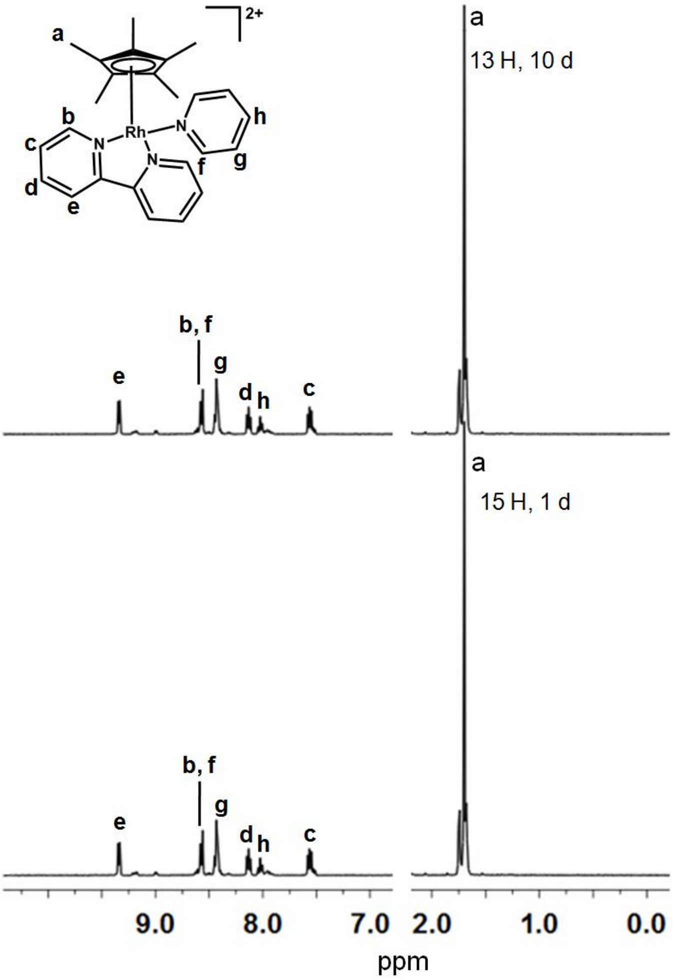

Figure S7. 400 MHz ^{1}H NMR spectrum of complex [(Cp*)Rh(bpy)(py)]$(PF_6)_2$ (**12**) in 60% MeOD-d_4/40% D_2O after incubation at 310 K for (a) 1 d, and (b) 10 d, showing the reduction in ring deuteration as a result of pyridine coordination.

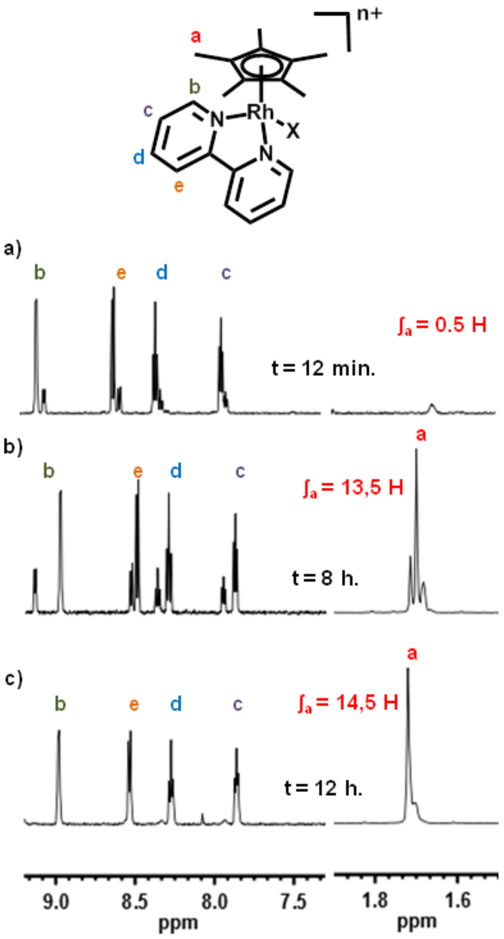

Figure S8. Effect of pH on deuteration of Cp* in complex **1**. ^{1}H NMR of complex **1** incubated in 60% MeOD-d$_4$/40% D_2O at different pH values. No silver nitrate was added to the reaction mixture. X = H_2O or Cl, n = 1 or 2. a) 12 min after addition of 50 µL of NaOD solution (40 wt% in D_2O, 99+ atom% D), integration of Cp* corresponds to 0 H (100% loss). b) After 8 h. No added acid or base, integration of the Cp* correspond to 13.5 H (10% loss). Two sets of peaks can be observed corresponding to the aqua and chlorido complexes. c) 12 h after addition of 50 µL of DNO_3 solution (65 wt % in D_2O, 99 atom% D), integration of Cp* corresponds to 14.5 H (0% loss compared to same sample recorded at t < 10 min).

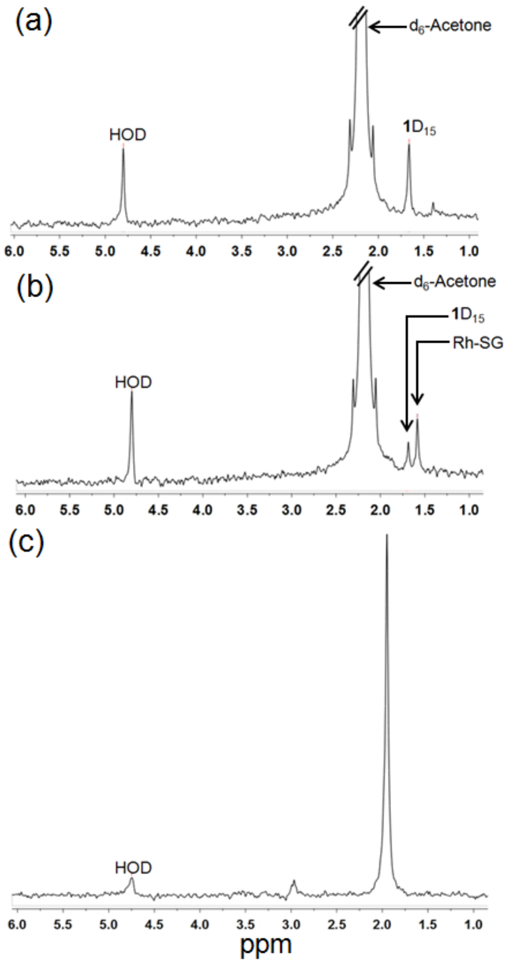

Figure S9. Examples of ^{2}H NMR (76.77 MHz) studies of reactions of fully deuterated complex D_{15}**1**. a) **1**D_{15} in 60% MeOH/40% H_2O containing d_6-acetone as internal standard. Back exchange, as monitored over time by the increase in intensity of the peak for HOD and decrease in intensity of the peak for **1**D_{15}, was slow (ca. 50% after 3 d at 310 K), as found also by MS. b) **1**D_{15} in 60% MeOH/40% H_2O in the presence of 0.75 mol equiv GSH showing formation of the complex [(Cp*)Rh(bpy)(GS)]. c) **1**D_{15}(3 mM) in RPMI-1640 cell culture medium supplemented with 10% of fetal calf serum. Interestingly the peak for **1**D_{15} is shifted to low field, suggesting interactions with components of the medium.

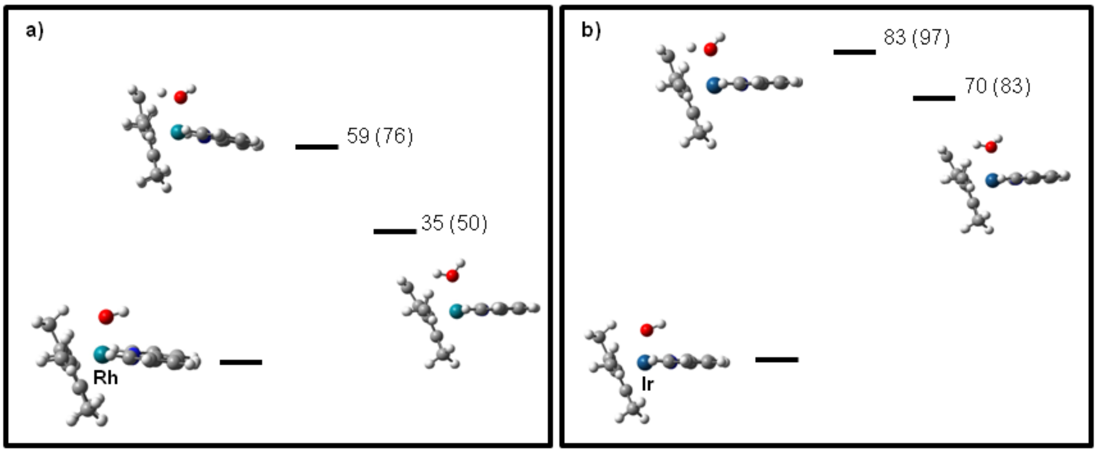

Figure S10. Energy profile for the deprotonation of CH_3 of Cp* calculated with DFT for isolated molecules. ΔH is in kJ/mol, the data in parentheses are those obtained for continuum polarization model (CPM) using methanol as solvent. a) $[Rh(bpy)(OH)Cp^*]^+$ The HO···H distances for the saddle point are 1.22 Å for the isolated molecule and 1.21 Å for the CPM model. The corresponding values of H_2C···H distances are 1.42 and 1.44 Å, respectively, and b) $[Ir(bpy)(OH)Cp^*]^+$ The HO···H distances for the saddle point are 1.17 Å for the isolated molecule and 1.16 Å for the CPM model. The corresponding values of H_2C···H distances are 1.50 and 1.52 Å, respectively.

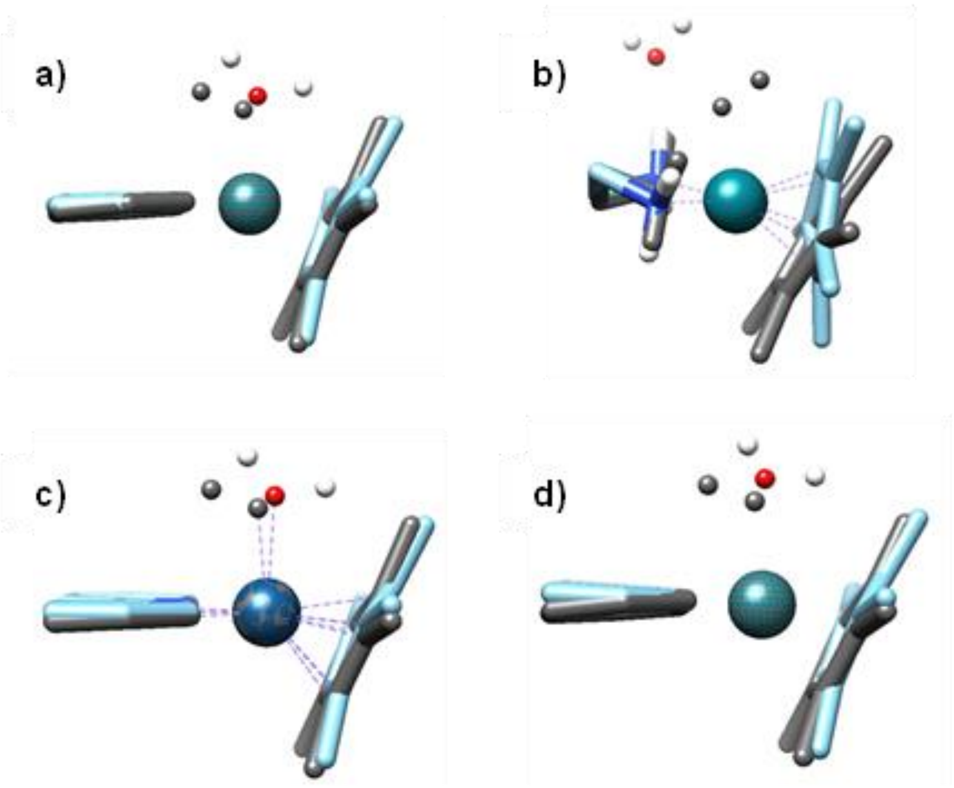

Figure S11. Superimposed optimized structures of the M(Cp^x)(*N,N'*)OH (in grey) and M([$Me_4Cp=CH_2$)(N,N')OH_2 (in color) complexes. a) M=Rh, (*N,N'*)=bpy; b) M=Rh, (*N,N'*)= en; c) M=Ir, (*N,N'*)=bpy; d) M=Rh, (*N,N'*)=bpy. Structures a, b and c were optimized with CAM-B3LYP/CEP-31G. Structure d was optimized with TPSSh/QZVP.

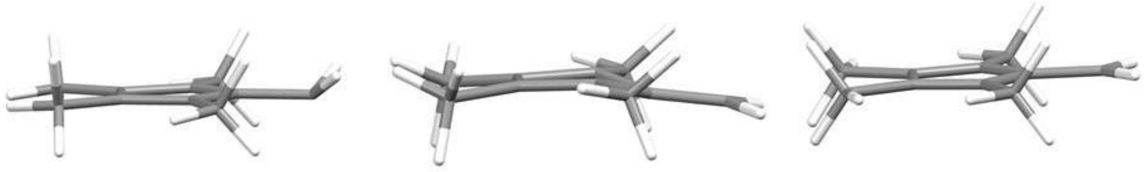

Figure S12. Comparison of a) optimized structure of $[Cp^*{=}CH_2]^{2-}$ (left), b) coordinated $Cp^*{=}CH_2$ fragment (center), and c) optimized structure of the neutral fulvene $Cp^*{=}CH_2$ (right). All calculations performed with CAM-B3LYP/CEP-31G method.

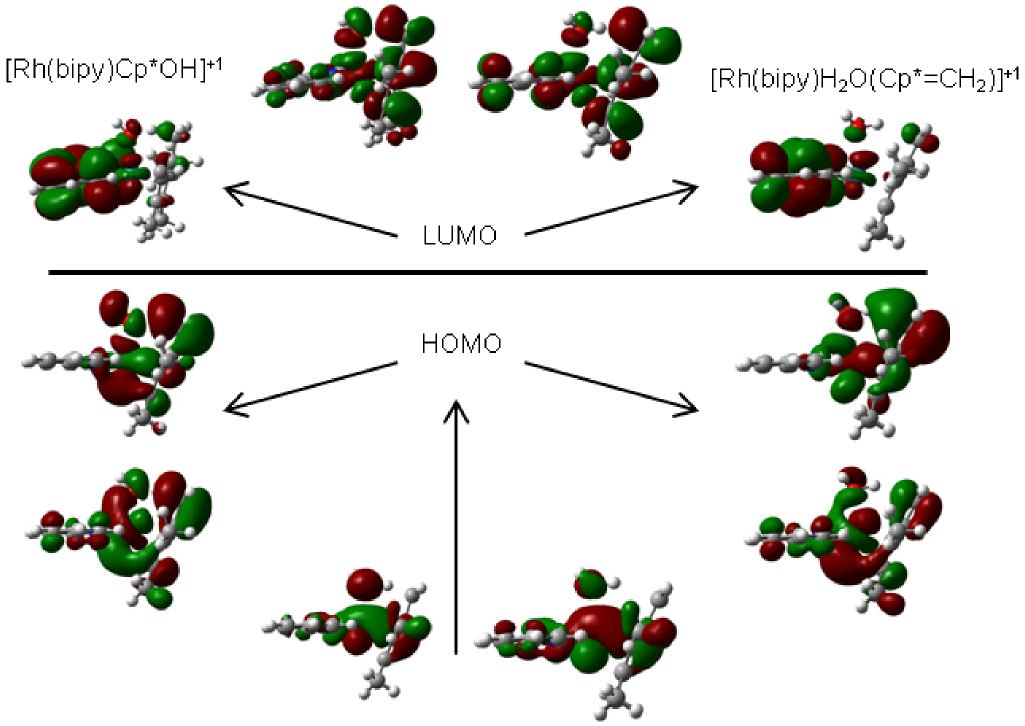

Figure S13. Occupied and virtual Kohn-Sham orbitals of pronounced d-character for $[(Cp^*)Rh(bpy)(OH)]^+$, and $[Rh(bpy)(H_2O)Cp^*{=}CH_2]^+$.

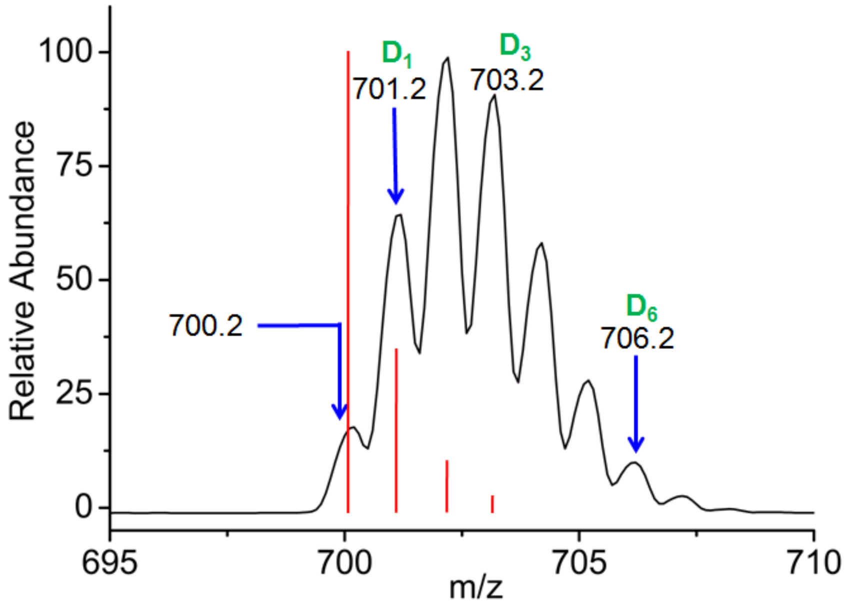

Figure S14. ESI-MS of a solution containing [(η^5-Cp*)Rh(bpy)(SG)]in D_2O formed from reaction of complex **1** with 1.5 mol equiv GSH after 72 h at 310 K, pH= 7.0; showing the various extents of deuteration. The theoretical isotopic distribution for non-deuterated complex [(η^5-Cp*)Rh(bpy)(SG)] is shown as red lines.

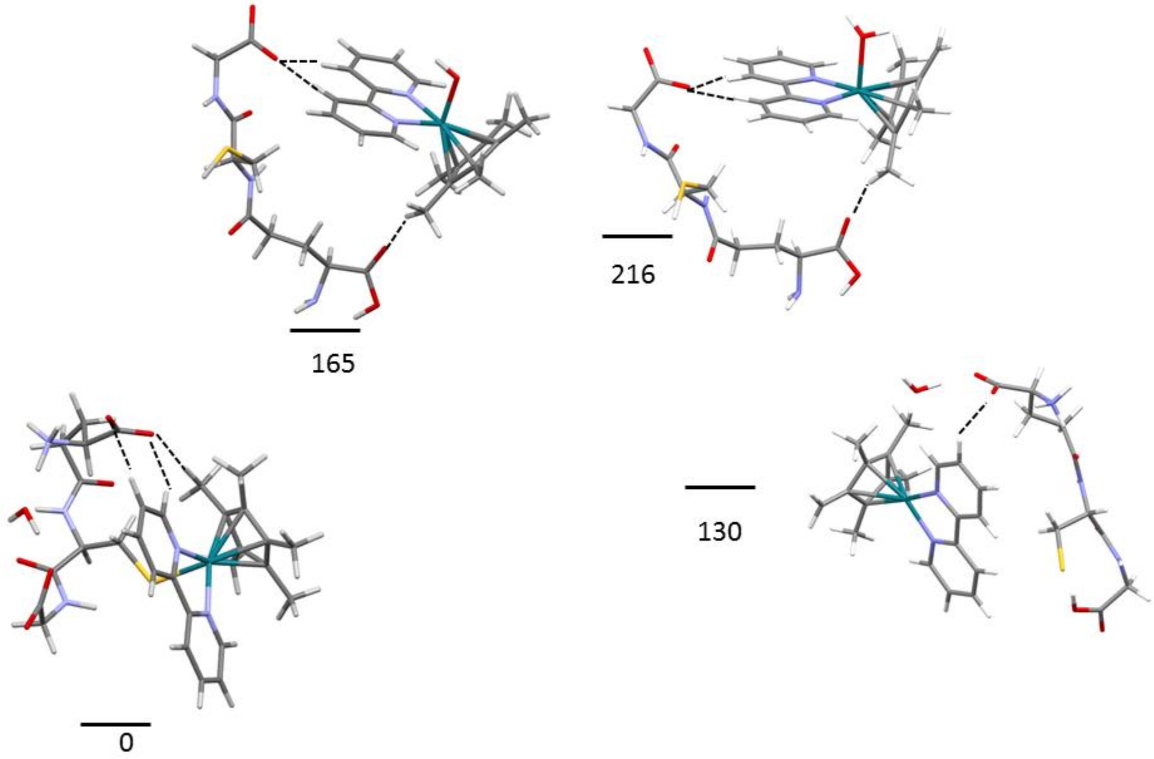

Figure S15. Optimized structures and relative energies (kJ/mol) of glutathione complexes and adducts of {(Cp*)Rh(bpy)} and its derivatives. Specific interactions are shown.

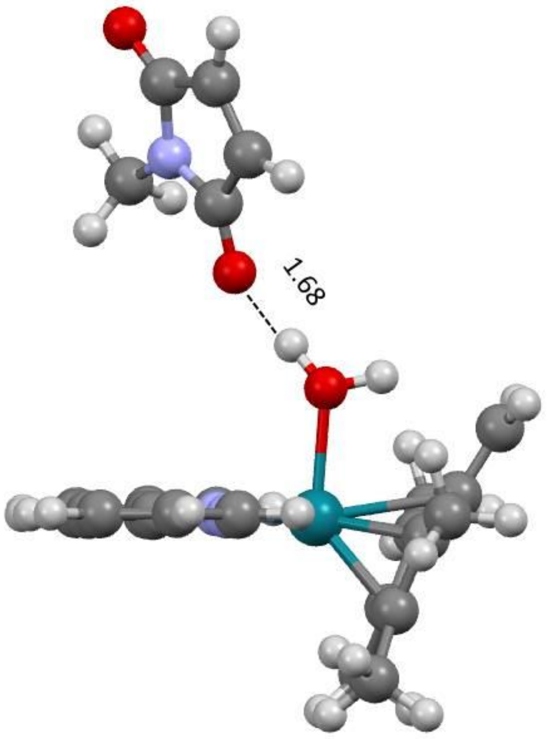

Figure S16. Optimised structure of the adduct of N-methyl maleimide and fulvene intermediate [Rh(bpy)(H_2O)(Me_4Cp=CH_2)]$^+$.

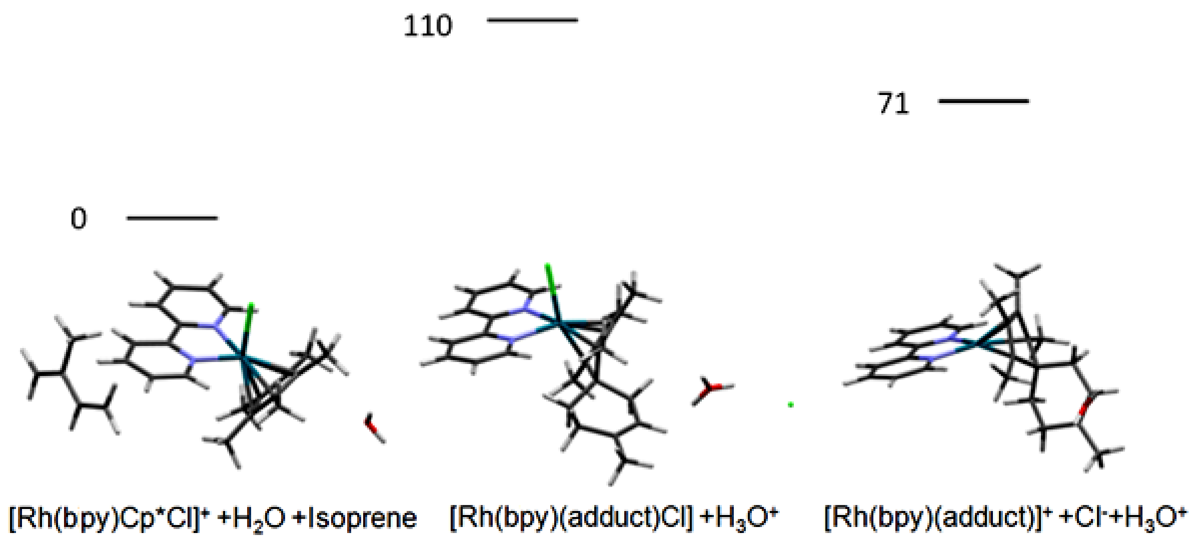

Figure S17. Comparison of the calculated ((CAM-B3LYP/CEP-31g, solvent = water, IEFPCM) electronic energies of the species along the pathway of the Diels-Alder reaction $[(Cp^*)Rh(bpy)Cl]^+$ + Isoprene + H_2O → $[Rh(bpy)(adduct)]^+$ + Cl^- +H_3O^+. The Rh-Cl bond lengths are 2.474 and 2.680 Å for the initial Cp* complex and the intermediate adduct, respectively. Adduct= tetramethyl fulvene + isoprene [2+4] cyclo-addition adduct.

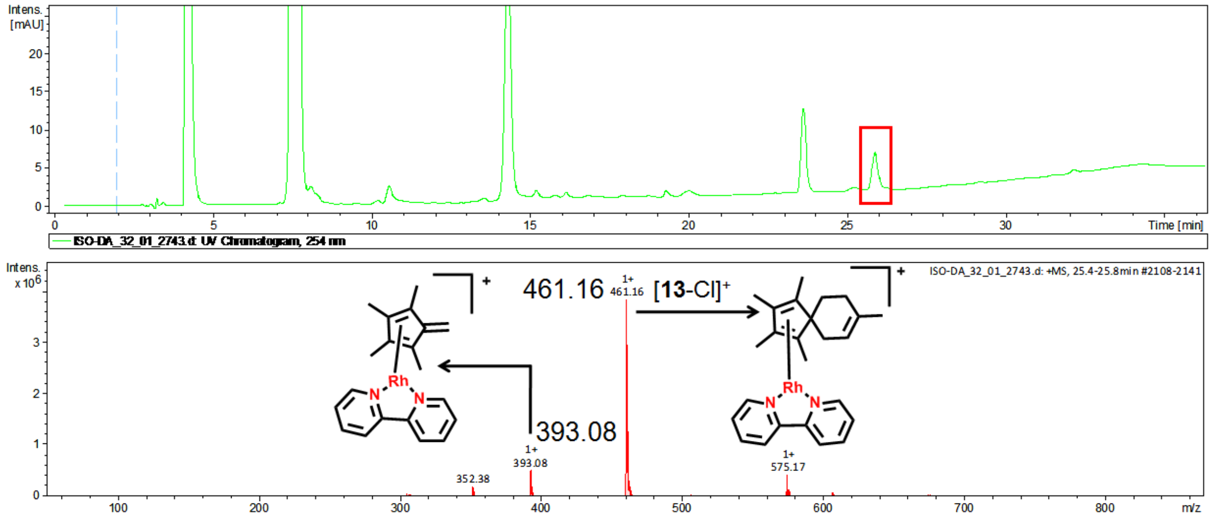

Figure S18. LC-MS characterization of the isoprene adduct, complex **13** in 60% MeOD-d_4/ 40% D_2O after 3 d of reaction of complex **1** with isoprene at 310 K.

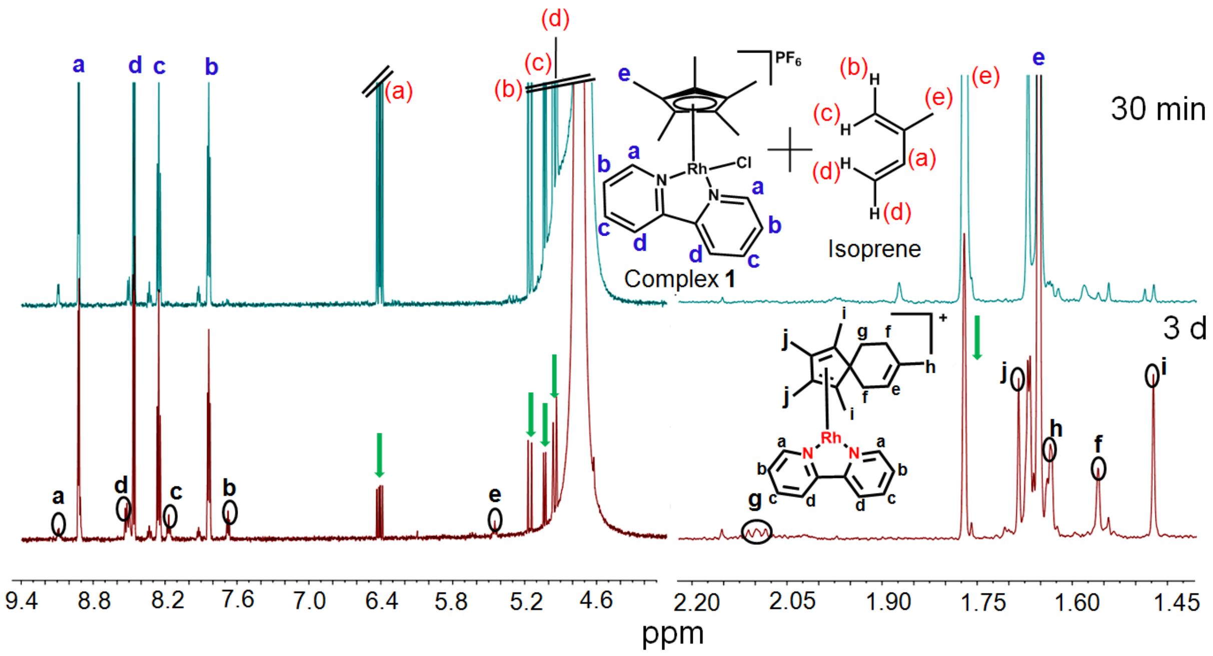

Figure S19. Time dependent 600 MHz ^{1}H NMR spectra for reaction of complex **1** with isoprene showing formation of the Diels-Alder complex **13** in 60% MeOD-d_4/40% D_2O after 3 days at 310 K. The decrease in the intensity of the isoprene peaks is indicated by green arrows. The new peaks which indicate the formation of the [4+2] cycloaddition adduct are assigned with integration in Figure 6.

.

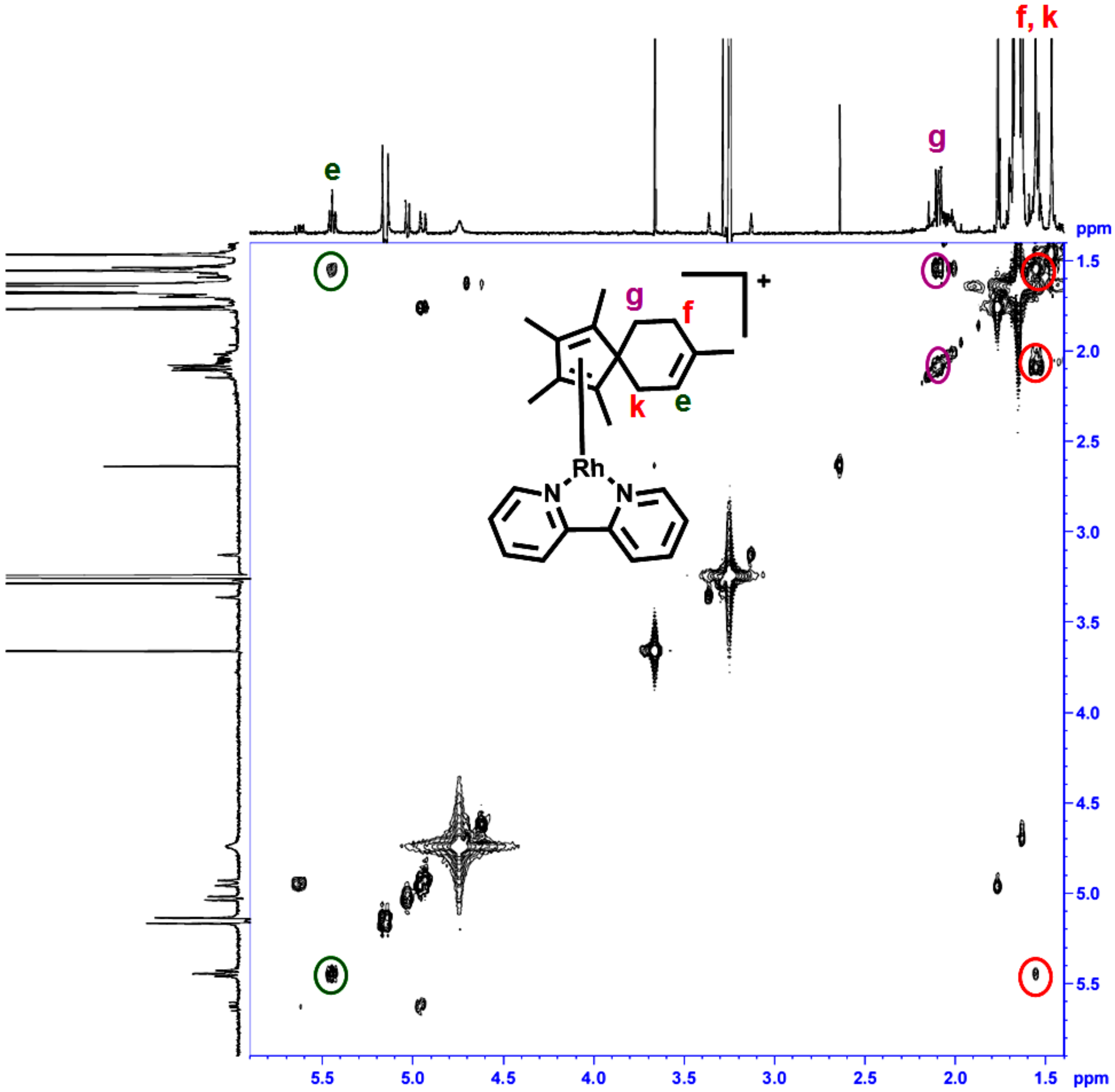

Figure S20. 2D ^{1}H NMR COSY spectrum of the Diels-Alder adduct of complex **1**with isoprene (complex **13)** in 60% MeOD-d$_4$/ 40% D$_2$O after 3 d of reaction at 310 K showing the 3-bond coupling of peak "f" (1.55 ppm) with peak "g" (2.10 ppm) and peak "k" (1.55 ppm) with that of "e" (5.43 ppm).

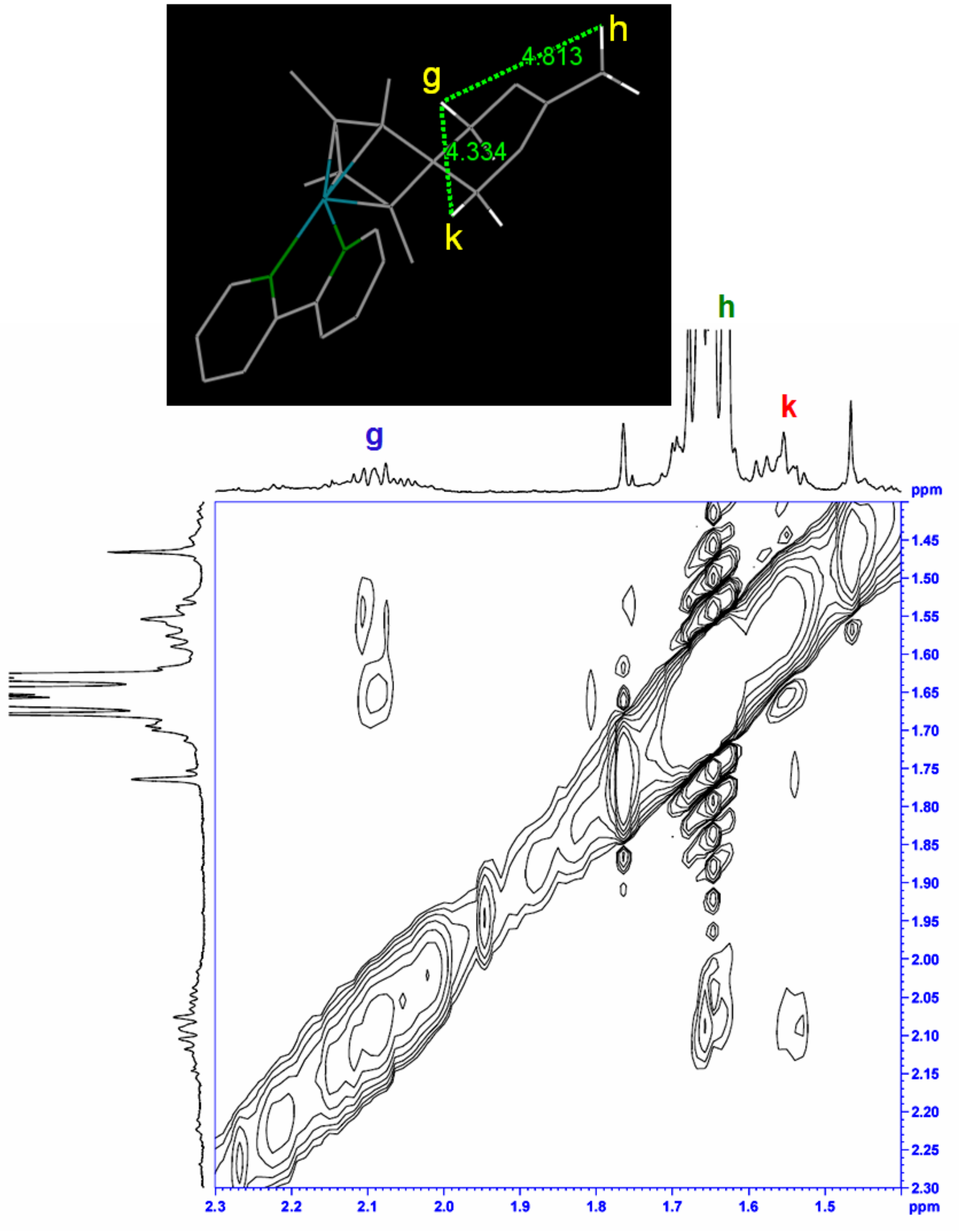

Figure S21. 2D ^{1}H NMR NOESY spectrum of the isoprene adduct (complex **13)** in 60% MeOD-d$_4$/ 40% D_2O for reaction of complex **1** and isoprene at 310 K showing the correlation of peak "g" (2.10 ppm) with peaks "h" (1.47 ppm) and "k" (1.55 ppm). The weak NOEs are consistent with the H-H distances of 4 – 5 À in the optimized structure (top inset).

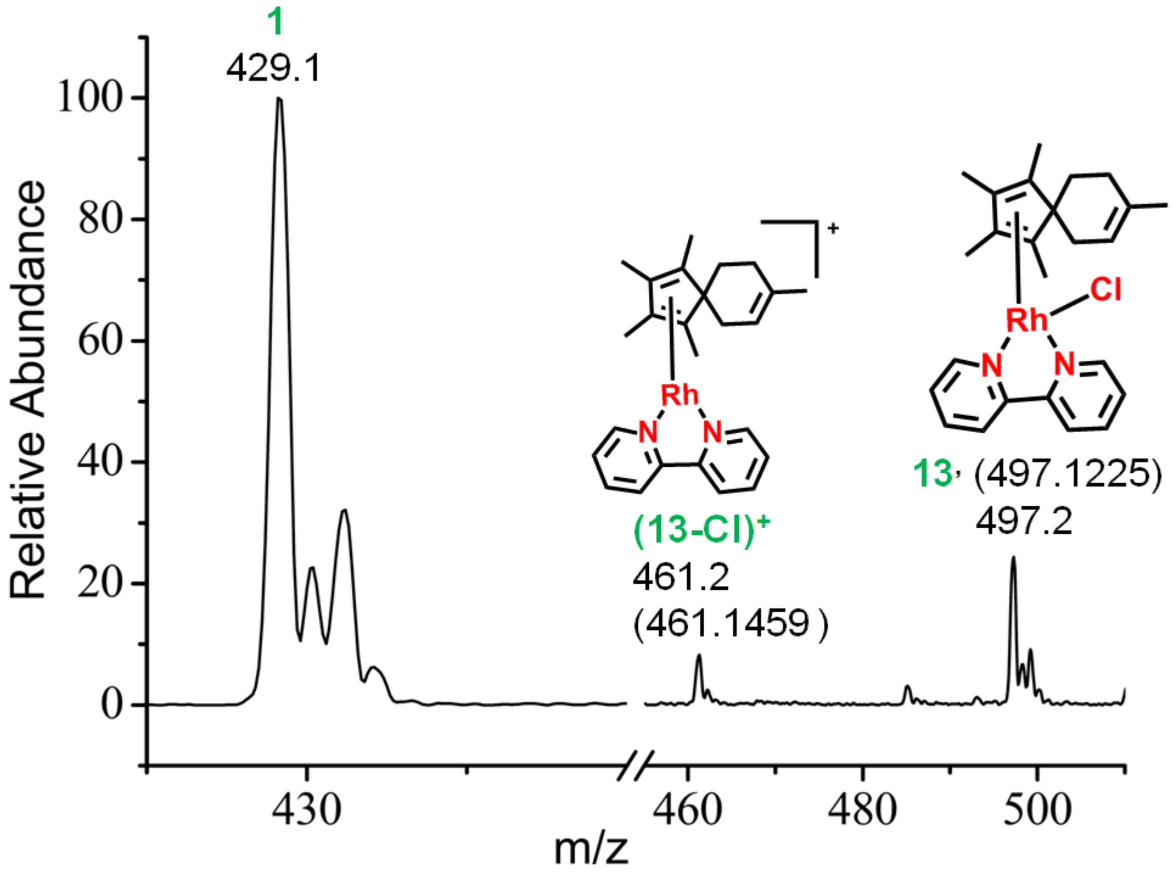

Figure S22. Mass spectrum of complex **1** after reaction with isoprene in RPMI-1640 cell culture medium supplemented with 10% of fetal calf serum showing the formation of the fulvene-isoprene [4+2] cyclo-addition adduct, complex **13** along with the typical isotopic distribution. RMPI-1640 contains several inorganic salts, amino acids, vitamins, glucose, and glutathione.

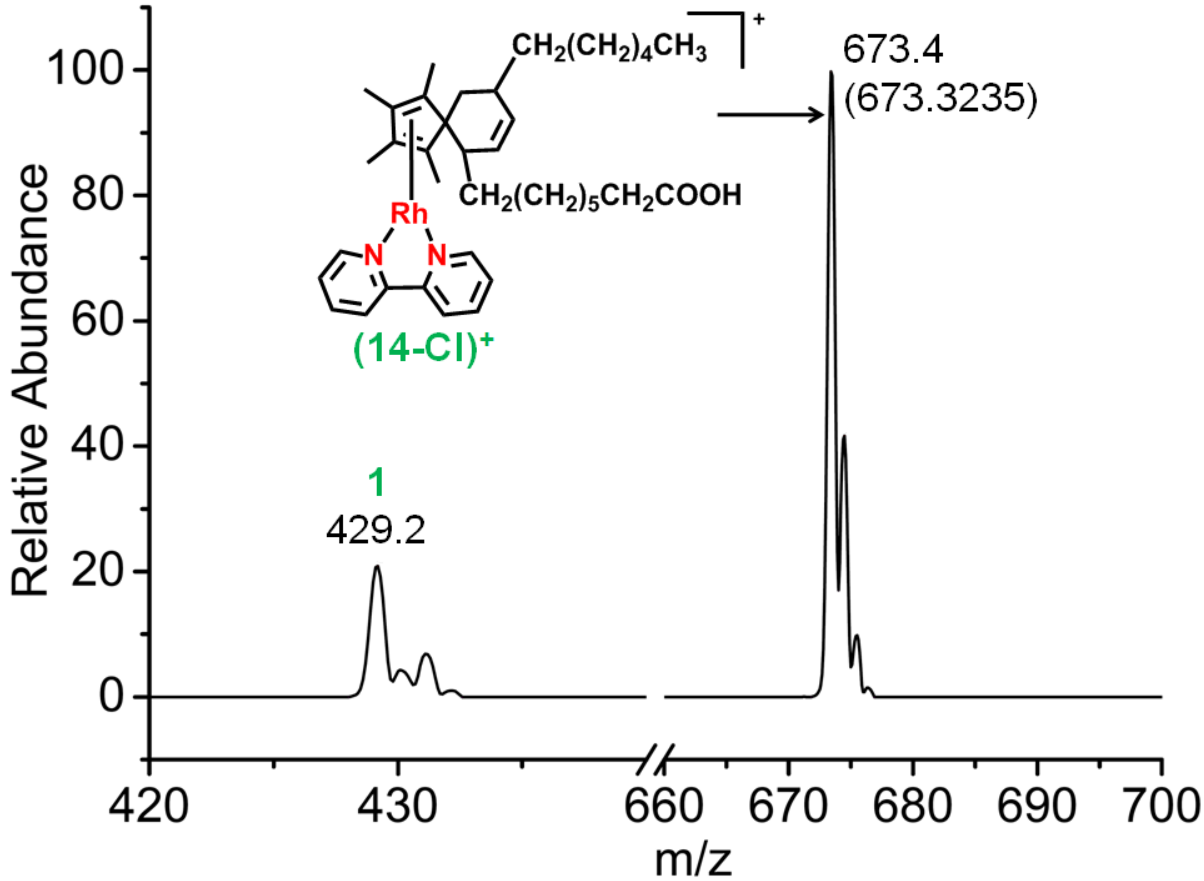

Figure S23. Mass spectrum of complex **1** after reaction with conjugated fatty acid, (9Z,11E)-linoleic acid in RPMI-1640 cell culture medium supplemented with 10% of fetal calf serum showing the formation of the fulvene-linoleic acid [4+2] cyclo-addition adduct, complex **14** along with its isotopic distribution.

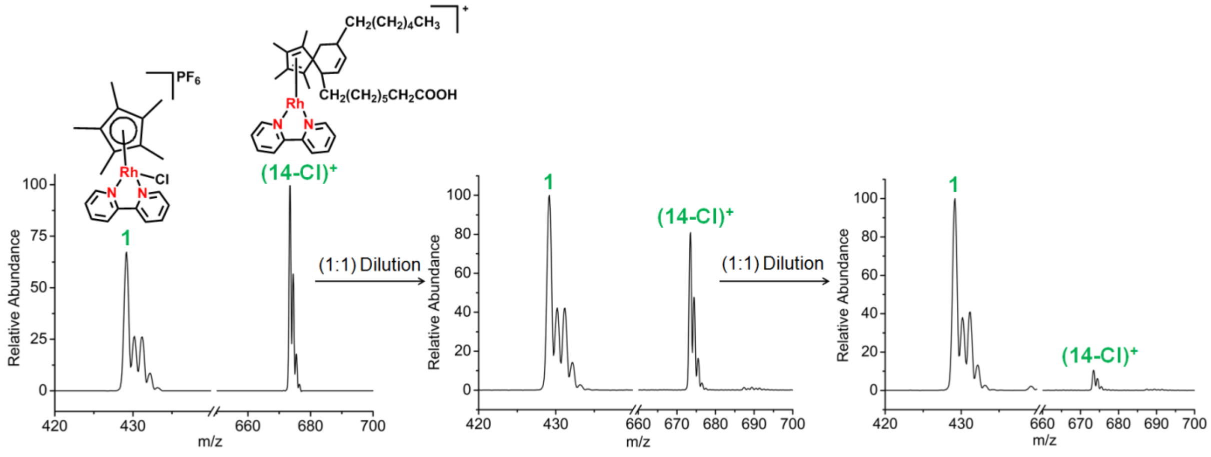

Figure 24. ESI-MS of a reaction mixture containing the Diels-Alder adduct of conjugated fatty acid, (9Z,11E)-linoleic acid with complex **1** (complex **14**) showing its dissociation on dilution (with 60% MeOD-d_4/ 40% D_2O), indicative of reversibility.

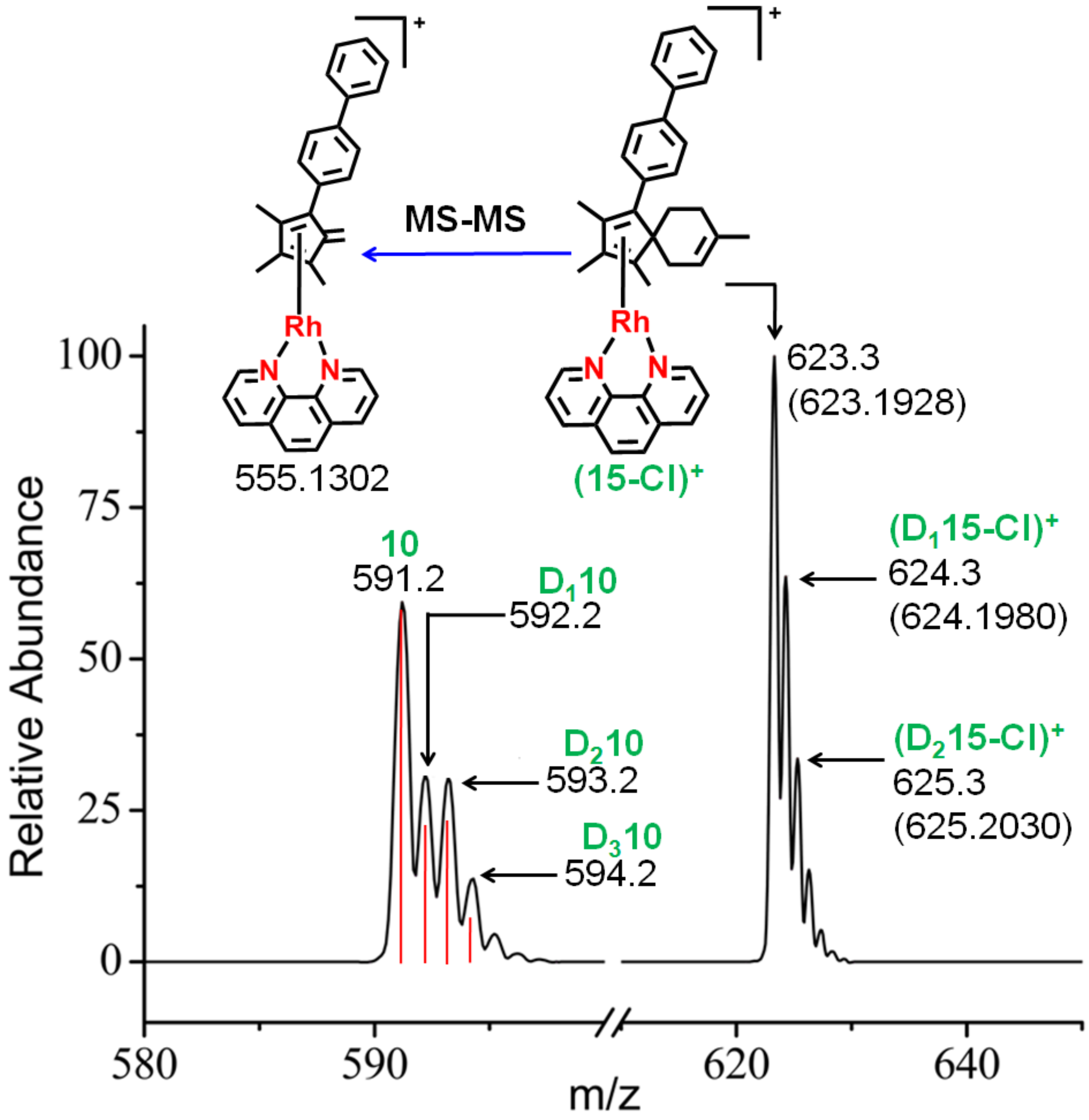

Figure S25. Mass spectra of complex **10** after reaction with isoprene showing the formation of the fulvene-isoprene [4+2] cyclo-addition adduct, complex **15**, along with deuteration of complex **10**. The theoretical isotopic distribution for non-deuterated complex **10** is shown as red lines.

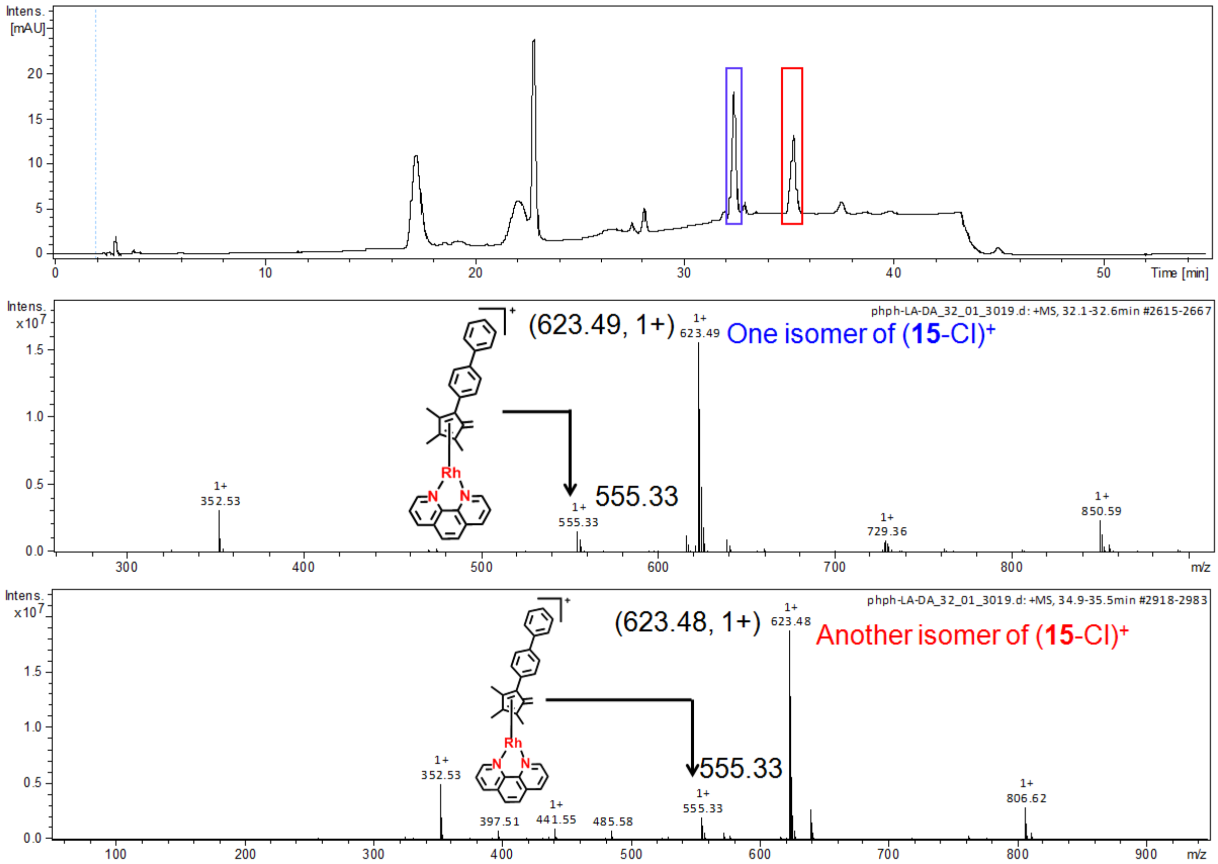

Figure S26. LC-MS characterization of the isoprene adduct, complex **15**, in 60% MeOD-d_4/ 40% D_2O after 3 d of reaction of complex **10** with isoprene at 310 K, indicating formation of the isomeric products.

References

1 M. Björgvinsson, S. Halldorsson, I. Arnason, J. Magull and D. Fenske, *J. Organomet. Chem.*,1997, **544**, 207-215.

2 Z. Liu, A. Habtemariam, A. M. Pizarro, S. A. Fletcher, A. Kisova, O. Vrana, L. Salassa, P. C. A. Bruijnincx, G. J. Clarkson, V. Brabec and P. J. Sadler, *J. Med. Chem.*,2011, **54**, 3011-3026.

3 G. M. Sheldrick, *SHELXL97 Program for Crystal Structure Refinement.*, University of Gottingen, Gottingen, 1997.

4 G. Sheldrick, *Acta Crystallographica Section A* 2008, **64**, 112-122.

5 O. V. Dolomanov, L. J. Bourhis, R. J. Gildea, J. A. K. Howard and H. Puschmann, *J. Appl. Cryst.*,2009, **42**, 339-341.

6 G. M. Sheldrick, *Acta Cryst.A*, 2015, **71**, 3-8.

7 G. M. Sheldrick, *Acta Cryst.C*, 2015, **71**, 3-8

8 Gaussian 09, Revision D.01,

M. J. Frisch, G. W. Trucks, H. B. Schlegel, G. E. Scuseria, M. A. Robb, J. R. Cheeseman, G. Scalmani, V. Barone, B. Mennucci, G. A. Petersson, H. Nakatsuji, M. Caricato, X. Li, H. P. Hratchian, A. F. Izmaylov, J. Bloino, G. Zheng, J. L. Sonnenberg, M. Hada, M. Ehara, K. Toyota, R. Fukuda, J. Hasegawa, M. Ishida, T. Nakajima, Y. Honda, O. Kitao, H. Nakai, T. Vreven, J. A. Montgomery, Jr., J. E. Peralta, F. Ogliaro, M. Bearpark, J. J. Heyd, E. Brothers, K. N. Kudin, V. N. Staroverov, T. Keith, R. Kobayashi, J. Normand, K. Raghavachari, A. Rendell, J. C. Burant, S. S. Iyengar, J. Tomasi, M. Cossi, N. Rega, J. M. Millam, M. Klene, J. E. Knox, J. B. Cross, V. Bakken, C. Adamo, J. Jaramillo, R. Gomperts, R. E. Stratmann, O. Yazyev, A. J. Austin, R. Cammi, C. Pomelli, J. W. Ochterski, R. L. Martin, K. Morokuma, V. G. Zakrzewski, G. A. Voth, P. Salvador, J. J. Dannenberg, S. Dapprich, A. D. Daniels, O. Farkas, J. B. Foresman, J. V. Ortiz, J. Cioslowski, and D. J. Fox, Gaussian, Inc., Wallingford CT, 2013.

9 T. Yanai, D. P. Tew and N. C. Handy, *Chem. Phys. Lett.*, 2004, **393**, 51-57.

10 W. J. Stevens, H. Basch and M. Krauss, *J. Chem. Phys.*,1984, **81**, 6026-6033.

11 W. J. Stevens, M. Krauss, H. Basch and P. G. Jasien, *Can. J. Chem.*, 1992, **70**, 612-630.

12 T. R. Cundari and W. J. Stevens, *J. Chem. Phys.*, 1993, **98**, 5555-5565.

13 J. P. Perdew, J. Tao, V. N. Staroverov and G. E. Scuseria, *J. Chem. Phys.*, 2004, **120**, 6898-6911.

14 A. Schäfer, C. Huber and R. Ahlrichs, *J. Chem. Phys.*, 1994, **100**, 5829-5835.

15 A. Schäfer, H. Horn and R. Ahlrichs, *J. Chem. Phys.*, 1992, **97**, 2571-2577.

16 M. C. Lehman, J. B. Gary, P. D. Boyle and M. S. Sanford, E. A. Ison, *Catalysis* 2013, **3**, 2304-2310.

17 G. Ciancaleoni, S. Bolano, J. Bravo, M. Peruzzini, L. Gonsalvi and A. Macchioni, *Dalton Trans.*, 2010, **39**, 3366-3368.
